# Solar Reforming of Biomass with Homogeneous Carbon Dots

**DOI:** 10.1002/anie.202008217

**Published:** 2020-09-01

**Authors:** Demetra S. Achilleos, Wenxing Yang, Hatice Kasap, Aleksandr Savateev, Yevheniia Markushyna, James R. Durrant, Erwin Reisner

**Affiliations:** ^1^ Christian Doppler Laboratory for Sustainable SynGas Chemistry Department of Chemistry University of Cambridge Lensfield Road Cambridge CB2 1EW UK; ^2^ Molecular Sciences Research Hub and Centre for Processable Electronics Imperial College London White City Campus London W12 0BZ UK; ^3^ Department of Colloid Chemistry Max Planck Institute of Colloids and Interfaces Research Campus Golm 14424 Potsdam Germany; ^4^ Present address: School of Chemistry University College Dublin Science Centre South, Belfield Dublin Ireland

**Keywords:** biomass, carbon dots, hydrogen, organics, photoreforming

## Abstract

A sunlight‐powered process is reported that employs carbon dots (CDs) as light absorbers for the conversion of lignocellulose into sustainable H_2_ fuel and organics. This photocatalytic system operates in pure and untreated sea water at benign pH (2–8) and ambient temperature and pressure. The CDs can be produced in a scalable synthesis directly from biomass itself and their solubility allows for good interactions with the insoluble biomass substrates. They also display excellent photophysical properties with a high fraction of long‐lived charge carriers and the availability of a reductive and an oxidative quenching pathway. The presented CD‐based biomass photoconversion system opens new avenues for sustainable, practical, and renewable fuel production through biomass valorization.

## Graphical abstract

Biomass‐derived materials photoconvert biomass: A triple natural resource system is reported that uses solar energy to convert biomass from untreated water into sustainable H_2_ and organics. The process is photocatalyzed by scalable carbon dots produced from α‐cellulose that act as water‐soluble light absorbers to photoconvert lignocellulose with a Ni cocatalyst. The carbon dots function under ambient conditions and benign aqueous solution.

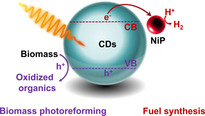

Photocatalysis allows for the utilization of solar energy to produce renewable H_2_, but most reported systems still require precious‐metal components, purified water or an expensive sacrificial electron donor (ED).^1^ Photoreforming (PR) can use sunlight to convert biomass waste into H_2_ and organic chemicals.^2^ Instead of oxidizing water as in classical artificial photosynthesis,^3^ PR employs preferentially abundant and inedible lignocellulose as an ED to quench holes (h^+^) in a photoexcited photocatalyst, leaving behind low‐potential electrons to drive proton reduction.^4^

PR commonly relies on UV‐absorbing TiO_2_ colloids with noble metal cocatalysts (Pt, RuO_2_),^5^ and toxic CdS in organic solvents (CH_3_CN)^6^ or alkaline conditions (pH>14).^7^ Carbon nitride (CN_*x*_) has been shown for visible‐light driven PR of biomass under benign aqueous pH,^8^ but the heterogeneous nature of CN_*x*_ restricts effective substrate/photocatalyst interactions to occur.2b, 6 Previous PR systems have also shown conversion yields ≤22 % (under strongly alkaline conditions) and required purified water,^5^, ^6^, ^7^, ^8^ which limit their utility, sustainability and economics.

Here, we introduce homogeneous carbon dots (CDs, Figure 1) produced from controlled, scalable calcination of cellulose (α‐*cel*‐CDs at 320 °C, Figure S1),^9^ or commercial precursors such as citric acid (resulting in amorphous CDs, *a*‐CDs at 180 °C, and graphitic CDs, *g*‐CDs at 320 °C),^10^ and aspartic acid (resulting in graphitic N‐doped CDs at 320 °C, *g*‐N‐CDs; see SI)10b, 11 for biomass PR. The non‐toxic, biocompatible CDs are employed as light absorbers, together with a Ni bis(diphosphine) H_2_ evolution cocatalyst (**NiP**,^12^ Figure S2), to produce H_2_ and organics in purified and untreated water under benign conditions (Figure 1 b). Transient absorption (TA) spectroscopy provides insight into the electron transfer dynamics of the PR systems.

**Figure 1:**
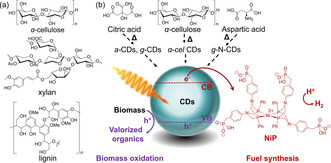
a) Chemical structures of lignocellulosic components used as EDs. b) CDs are synthesized from biomass (α‐cellulose) or commercial precursors (citric, aspartic acid) and used with **NiP** as cocatalyst in PR of biomass to coproduce H_2_ and oxidized organics.

α‐*cel*‐CDs (diameter: 9±3 nm) and *g*‐N‐CDs (3±1 nm) are graphitic with (100) intralayer spacings of 3.0 and 2.4 Å, respectively.^9^, 10b Powder XRD also suggests nanocrystalline, low defect graphitic structures for α‐*cel*‐CDs (27.6° 2*θ)* and *g*‐N‐CDs (27.0° 2*θ*), in agreement with Raman (graphitic content, G band, 1570–1580 cm^−1^ and defects, D band, 1331–1340 cm^−1^) and ^13^C NMR spectroscopy (predominant sp^2^ environments, *δ*=110–180 ppm, no sp^3^ centers).^9^, 10b *g*‐CDs (4±1 nm) are graphitic, whereas *a*‐CDs (7±2 nm) are amorphous.^10^

Photocatalysis with CDs (0.03–2.8 mg) and **NiP** (50 nmol) was first performed using ethylenediaminetetraacetic acid (EDTA, 0.1 m, pH 6) as the sacrificial ED in purified water (3 mL, Figure 2 a, S3). All systems were irradiated with simulated solar light (AM 1.5G, 100 mW cm^−2^) under an inert atmosphere at 25 °C and the headspace gas was analyzed by gas chromatography. H_2_ yields (in μmol, Figure 2 a) and specific activities (μmol H_2_ (g_CDs_)^−1^ h^−1^, Figure S3, Tables S1–S4) were optimized by varying the amounts of CDs. α‐*cel*‐CDs showed consistently the highest H_2_ yields and their best performance at 2.2 mg (15.6±0.7 μmol H_2_, 24 h, Figure 2 a). The α‐*cel*‐CDs/**NiP** system was also photocatalytically active under visible‐light only irradiation (*λ*>400 nm), albeit with a lower H_2_ yield (28 %). CDs have sufficient driving force for proton reduction (CB at approximately −0.5 V vs. RHE),^13^ however, the accurate determination of their band levels is crucial for their future development as photocatalysts.

**Figure 2:**
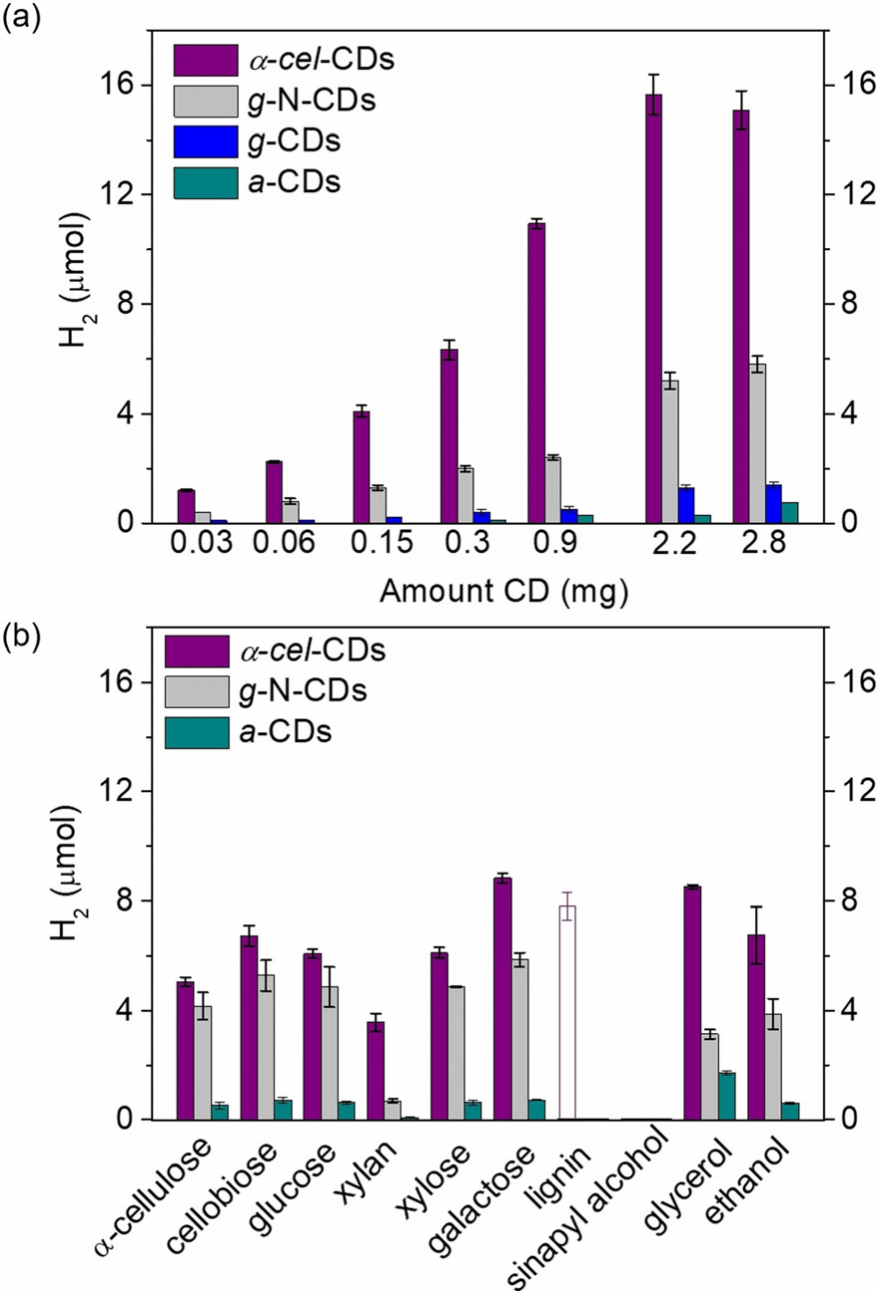
a) Photo‐H_2_ evolution using α‐*cel*‐CDs, *g*‐N‐CDs, *g*‐CDs, and *a*‐CDs (0.03–2.8 mg) and EDTA (0.1 m, pH 6, 3 mL) as a sacrificial ED. b) Photo‐H_2_ evolution with α‐*cel*‐CDs (2.2 mg), *g*‐N‐CDs (0.5 mg), and *a*‐CDs (10 mg) using pure lignocellulosic components and soluble substrates (100 mg, solid bars) in purified water (KP_i_, pH 6). The empty bar shows the result using 0.5 mg of lignin. Conditions: AM 1.5G (100 mW cm^−2^) irradiation, with **NiP** (50 nmol) for 24 h and 25 °C.

The α‐*cel*‐CD/**NiP** system provides a benchmark activity of 13 450 μmol H_2_ (g_CDs_)^−1^ h^−1^ (Figure S3, Table S5).^10^, ^11^, ^14^ The α‐*cel*‐CDs display a maximum internal quantum efficiency (IQE at *λ*=360 nm, *I*=4.05 mW cm^−2^) of 11.4 %, which compares favorably with *g*‐N‐CDs (5.3 %) and *a*‐CDs (1.4 %).10b Future improvements in the development of the CDs should focus on high IQEs in the visible region. The photo‐stability of the CD/**NiP** systems is currently limited by the fragile ligand framework of **NiP**, which degrades after a few hours of operation either due to formation of radicals from EDTA oxidation or ligand displacement from the Ni center.^13^ 4‐Methylbenzyl alcohol (30 μmol) instead of EDTA produced 3.7±0.2 μmol H_2_ after 6 h irradiation with α‐*cel*‐CD/**NiP** (Figure S4, Table S6).

We then studied various insoluble biomass (α‐cellulose, xylan and lignin; Figure 1 a) and soluble biomass model substrates and alcohols of industrial relevance (ethanol, glycerol; Figure S5). PR in aqueous phosphate solution (KP_i_; pH 6 and 25 °C) with the CDs showed activity under benign conditions (Figures 2 b, S6, Tables S6–S9), with the α‐*cel*‐CDs showing again the best activity (Figures 2 b).

The highest H_2_ yields after 24 h were observed with galactose (8.8±0.2 μmol) and glycerol (8.5±0.1 μmol), which correspond to turnover numbers of **NiP** (TON_**NiP**_) of 177±4 and 170±2, respectively. Control experiments without ED, CDs or **NiP** showed negligible or no H_2_ evolution (Figure S7 and Table S7). The lowest H_2_ yields were observed for lignin (0.03 μmol) due to its strong light absorption and robust cross‐linked polyphenolic structure.^15^ However, a much higher H_2_ yield (7.8±0.5 μmol, Table S6) was observed at lower lignin quantities (0.5 mg) due to improved light penetration through the CD solution (Figure 2 b, empty bar). PR of α‐cellulose and xylan produced 5.0±0.2 and 3.6±0.3 μmol H_2_, respectively, similar to a heterogeneous CN_*x*_/**NiP** system.8a However, in contrast to heterogeneous systems that show substrate‐dependent H_2_ yields, homogeneous CDs photoreform soluble and insoluble biomass with a similar efficiency.

PR of α‐cellulose with the α‐*cel*‐CD/**NiP** system was subsequently studied in KP_i_ (pH 4.5, 6 and 8), H_2_SO_4_ (pH 2) and 10 m KOH (≈pH 15) (Figure S8). The highest H_2_ yields after 24 h were observed at pH 6 (5.0±0.2 μmol H_2_) and pH 8 (3.6±0.2 μmol H_2_). The efficiency was decreased approximately four times (1.2±0.1 μmol H_2_) in strong acid (pH 2), and PR did not proceed under extremely basic conditions (10 m KOH) due to the chemical instability of **NiP** (Figure S8 and Table S10).^13^

The biomass conversion yield (CY, %) was determined in KP_i_ pH 6 with α‐*cel*‐CD/**NiP** at various α‐cellulose loadings (0.8–1.65 mg, Figure S9, Table S11). A CY of 13.4 % was achieved at 0.8 mg α‐cellulose (12 hrs), whereas re‐additions of **NiP** (50 nmol) to repair the PR system in situ allowed a CY of 34.1 % (48 h, Figure S9).^13^ This is higher than CYs reported for CdS/CdO_*x*_ (9.7 %)^7^ and CN_*x*_/Pt (22 %)8a under strongly alkaline conditions.

The oxidation products were determined by High Performance Liquid Chromatography Mass Spectrometry (HPLC/MS) and ^1^H, ^13^C NMR spectroscopy after PR of α‐cellulose, xylan, glucose and galactose with α‐*cel*‐CDs (2.2 mg) and **NiP** (50 nmol) in KP_i_ (pH 6; see Figures S10–S17 for detailed analysis). In brief, the main products of α‐cellulose PR are C_6_H_12_O_6_ and C_6_H_10_O_5_ compounds (e.g., 2,5‐anhydro‐d‐mannofuranose isomers). HPLC/MS and ^13^C NMR spectroscopy suggest the formation of 2,3,4,5,6‐pentahydroxyhexanoate along with other oligosaccharides after PR of uniformly ^13^C‐labeled cellulose. PR of xylan produced hydroferulic acid C_10_H_12_O_4_/C_11_H_14_O_4_ derivatives and other depolymerization products. PR of galactose/glucose resulted in C_6_H_12_O_6_ and C_6_H_10_O_5_ isomers.

PR of α‐*cel*‐CDs (2.2 mg) with biomass substrates (100 mg) was then studied in untreated sea water (adjusted pH 6; Figures S18, S19, Tables S12–S14). The H_2_ yields are comparable to purified water as reaction medium, suggesting that impurities/background organics do not hinder photocatalysis as observed for TiO_2_‐based systems, but may rather act as EDs.^16^ The highest H_2_ yields were again achieved with galactose (8.4±0.1 μmol, 24 h). The *g*‐N‐CDs showed 2–7 times lower H_2_ yields in sea water compared to purified water (≤2.3±0.1 μmol, 24 h), presumably due to surface N‐doping that may provide adsorption sites for contaminants from the impurity‐rich water.16a *a*‐CDs in sea water show low H_2_ yields (≤0.3 μmol), comparable to purified water. Thus, undoped CDs maintain good photocatalytic performances under real‐world conditions.16a

TA spectroscopy was employed to study the photophysics and charge transfer properties of α‐*cel*‐CDs, on fs‐ns (fs‐TA) and μs‐s (μs‐TA) timescales. fs‐TA spectra (355 nm excitation, under Ar) resulted in a broad absorption feature in the visible region (Figure S20), which decays ≈2 fold faster upon adding EDTA, with the decay halftime changing from ≈20 to 40 ps (Figure 3 a). This indicates that the absorption contains a partial contribution from photoinduced h^+^ that are scavenged by EDTA (≈0.1 ns),8c, 16c most likely by pre‐adsorbed ED species.

**Figure 3:**
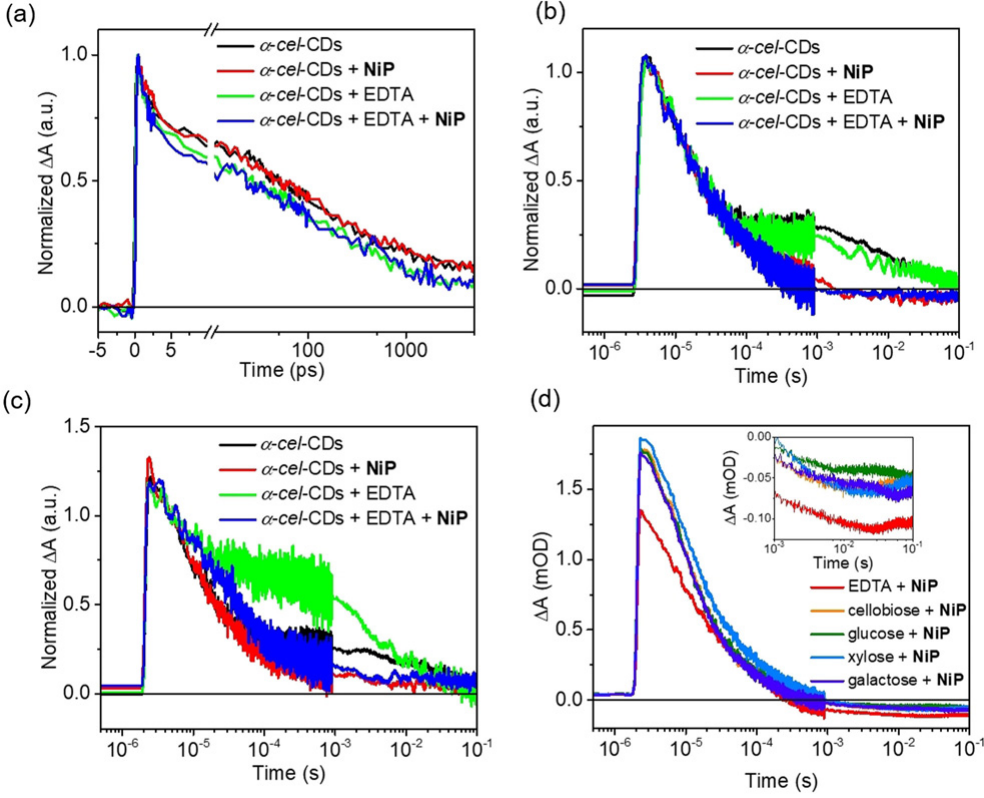
Normalized a) (≈1 ps) fs‐TA kinetics between 500 and 520 nm, b) (≈50 μs) μs‐TA kinetics (electrons) at 500 nm, c) (≈50 μs) μs‐TA kinetics (electrons) at 700 nm of α‐*cel*‐CDs with EDTA and/or **NiP**. d) Normalized (≈50 μs) μs‐TA kinetics (electrons) of α‐*cel*‐CDs at 500 nm with **NiP** and various biomass EDs (0.1 m). Inset shows the bleach region of ΔA which corresponds to **NiP^−^**. Conditions: KP_i_ (pH 6.6) with **NiP** (50 nmol) upon excitation at 355 nm with an energy of 1 mJ cm^−2^.

On μs‐s timescales, a blue‐shifted, long‐lived signal is observed in the absence of EDTA (Figure S21), which is effectively quenched by O_2_ and thus originates primarily from electrons. These are long‐lived, trapped charge carriers with residual signals (≈100 ms) even without EDTA, similar to previous reports for C_3_N_4_,^17^ and metal oxide photocatalysts.^18^ Addition of **NiP** as electron scavenger for α‐*cel*‐CDs resulted in *(i)* quenching of the electron signal (≈0.5 ms) and *(ii)* appearance of a negative signal, assigned to the ground‐state bleach of **NiP** due to its reduction by CDs, at 500 nm (Figures 3 b, S22).10b, 12, 19 This suggests the direct electron transfer from CDs* to **NiP**, even without EDTA, therefore demonstrating an oxidative quenching mechanism. Titration of CDs with **NiP** (Figure S23) revealed a linear relationship between the electron decay rates (at 500 nm) and **NiP** concentration, and an oxidative quenching rate of 1.09±0.04×10^8^ m ^−1^ s^−1^. This mechanism will have a low overall yield, as without EDTA most electrons recombine on faster timescales (≪100 ms), consistent with negligible H_2_ production (Table S7). Nevertheless, the ability of long‐lived trapped electrons to reduce **NiP** indicates that they retain reactivity, with trap energies above the **NiP** reduction potential.

Consistent with the fast hole scavenging process (≈0.1 ns), addition of EDTA resulted in prolonged electron signals at 700 nm (Figure 3 c), indicative of reductive quenching. Signals at 500 nm were not prolonged with EDTA, suggesting multiple electronic states in α‐*cel*‐CDs.^20^ Nevertheless, these results show both oxidative and reductive quenching for α‐*cel*‐CDs, which is different from that observed for *g*‐N‐CDs and *a*‐CDs under similar conditions. In the latter cases, **NiP^−^** can only be formed with EDTA,10b most likely due to differences in energy of the trapped charges between these samples. For α‐*cel*‐CDs, the appearance of the **NiP^−^** signal at 500 nm at long times (Figures S22e) is indicative of reasonably efficient photoinduced **NiP** reduction (Figure 4).

**Figure 4:**
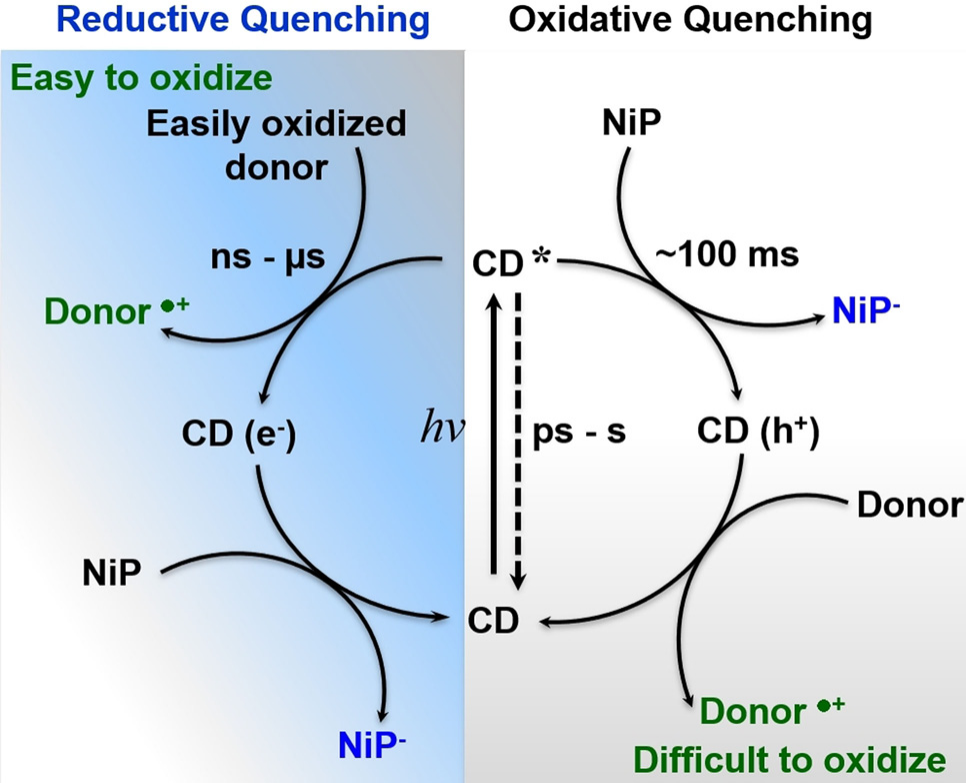
Timescales of relaxation and possible charge transfer reactions under photocatalytic conditions for α‐*cel*‐CDs.

Previous studies on *g*‐N‐CDs showed a bimolecular recombination lifetime of *t*_50 %_=9 ps, with a residual 6 % of long‐lived carriers (5 ns) to drive H_2_ production.10b Herein, using similar excitation fluence/buffer conditions, the α‐*cel*‐CD bimolecular recombination lifetime is *t*_50 %_=45±5 ps (i.e., 5 times slower), with the proportion of long‐lived (>5 ns) carriers being about 15–20 % (Figure 3 a). We can thus propose two reasons for improved photocatalysis with α‐*cel*‐CDs: (*i*) existence of both oxidative and reductive quenching mechanisms and (*ii*) α‐*cel*‐CDs show slower bimolecular recombination processes and higher yields of long‐lived carriers, which enable higher H_2_ yields both under model (Figure 2 a) and real‐world conditions (Figure S18).

Finally, μs‐TA spectra of α‐*cel*‐CDs with biomass were collected to analyze their capacity to quench the photogenerated h^+^. Biomass addition induced a similar oxidative quenching mechanism as with EDTA (Figures S24), but with a 50 % lower yield of **NiP^−^** (Figure 3 d). The slower h^+^ extraction is assigned to the less accessible biomass compared to EDTA, which results in increased recombination and thus fewer long‐lived electrons that can be extracted by **NiP**. This agrees with photocatalysis, where twice the H_2_ yield was observed with EDTA compared to biomass (Figure 2). It is also possible that long‐lived, trapped h^+^ accumulate in CDs with biomass as ED due to the oxidative quenching pathway by **NiP** (Figure 4, white panel), facilitating oxidation of the challenging lignocellulosic substrates.

In summary, we report the development of a homogeneous PR system using CDs as light absorbers, which use the nexus of natural resources for coupled sustainable fuel production with biomass utilization and chemical synthesis. CDs prepared from biomass have well‐suited photophysical characteristics such as the availability of an oxidative quenching pathway to convert challenging substrates and a high fraction of long‐lived charge carriers. The cellulose‐derived CDs allow for solar‐driven fuel synthesis from lignocellulosic biomass under benign conditions with the prospect to simultaneously produce valuable chemicals in solution. The PR systems operate with a noble‐metal‐free cocatalyst and maintain their photocatalytic activity even in untreated sea water, which creates promising perspectives for the development of energy self‐sufficient and low‐carbon economies.

## Conflict of interest

A patent application on this work has been filed by Cambridge Enterprise (WO/2019/229255), which lists three co‐authors (D.S.A., H.K. and E.R.) as inventors.

## Supporting information

As a service to our authors and readers, this journal provides supporting information supplied by the authors. Such materials are peer reviewed and may be re‐organized for online delivery, but are not copy‐edited or typeset. Technical support issues arising from supporting information (other than missing files) should be addressed to the authors.

Supplementary
