# Supplementary material for: Solar Reforming of Biomass with Homogeneous Carbon Dots
Source: Angew Chem Int Ed Engl. 2020 Sep 1;59(41):18184–8. doi: 10.1002/anie.202008217 (PMC7589312; doi:10.1002/anie.202008217)
Supplement: Supplementary file 1 — Supplementary [file ANIE-59-18184-s001.pdf]

Angewandte Chemie
Eine Zeitschrift der Gesellschaft Deutscher Chemiker

Supporting Information

Solar Reforming of Biomass with Homogeneous Carbon Dots

Demetra S. Achilleos, Wenxing Yang, Hatice Kasap, Aleksandr Savateev, Yevheniia Markushyna, James R. Durrant, and Erwin Reisner**

anie_202008217_sm_miscellaneous_information.pdf

Experimental Section

Materials. All reagents were purchased from Sigma Aldrich and used without further purification.

CD synthesis. *α-cel*-CDs were synthesized upon thermal treatment of *α*-cellulose (10 g) at 320 °C in air for 100 h and characterized as previously reported.[1] The material obtained from calcination was used for photocatalytic experiments without further treatment.

g-N-CDs were synthesized as reported previously.[2] In brief, aspartic acid (5 g) was calcinated at 320 °C for 100 h. Next, the sample was dissolved in water (100 mL) and NaOH was added (5 M, 200 *μ*L) to produce a dark brown solution, which was passed through a microfilter (0.22 *μ*m). *g*-N-CDs were isolated as a dark-brown solid upon freeze drying for 2 days.

g-CDs were synthesized via a two-step thermal process from citric acid; the precursor was treated thermally first at 180 °C for 40 h, followed by a second calcination step at 320 °C for 100 h.[2] The dark brown solid was dissolved in water (100 mL) bestowed with NaOH (5 M, 200 *μ*L), and passed through a microfilter (0.22 *μ*m). *g*-CDs were then freeze-dried for 2 days.

a-CDs were also synthesized from citric acid, but at a lower temperature than *g*-CDs; at 180 °C for 40 h in air.[3] The samples were neutralized with NaOH as described above and freeze-dried to isolate a readily water soluble yellow solid.

Nickel bis(diphosphine) (NiP) synthesis. The hydrogen evolution co-catalyst, **NiP**, was synthesized and characterized as bromide salt as reported previously.[4]

Photocatalytic experiments. The samples for photocatalytic experiments were prepared in borosilicate glass vials (7.7 mL). Initially, photocatalytic experiments were carried out under model conditions by dissolving 0.03–2.80 mg of *α-cel*-CDs, *g*-N-CDs, *g*-CDs and *a*-CDs in aqueous EDTA solutions (0.1 M, 3 mL, pH 6) in the presence of **NiP** catalyst. The vials were then sealed with Subaseal rubber septa, degassed for 20 min with N_2 containing 2% CH_4 (internal gas chromatography standard) and irradiated using a Newport Oriel solar light simulator (100 mW cm^{-2}) equipped with an air mass 1.5 global filter (AM 1.5G) and water filter to remove infrared irradiation. During irradiation the temperature was maintained at 25 °C and the samples were stirred constantly. Samples of the headspace gas (20 *μ*L) were taken from the photoreactor and analyzed by gas chromatography at an hourly basis for the first 6 h and then after 24 h. For this purpose, an Agilent 7890A gas chromatograph was used, equipped with a 5 Å molecular sieve column and a thermal conductivity detector (TCD).

Photocatalytic experiments with *α-cel*-CDs (2.2 mg) under visible-light irradiation only, were carried out in the presence of a longpass filter (λ > 400 nm) in EDTA solutions (0.1 M, 3 mL, pH 6) with **NiP** (50 nmol). When 4-methylbenzyl alcohol (4-MBA) was used as ED, 2.2 mg of *α-cel*-CDs were dissolved in KP_i (pH 6, 3 mL) in the presence of 30 *μ*mol of the alcohol.

To study biomass-based substrates as EDs, photocatalytic experiments were carried out under optimum conditions for each CD system. For this purpose, α-*cel*-CDs (2.2 mg), *g*-N-CDs (0.5 mg), *g*-CDs (0.5 mg) and *a*-CDs (10 mg) were dissolved in KP_i (pH 6, 3 mL) with 100 mg of the EDs (α-cellulose, cellobiose, glucose, xylan, xylose, galactose, lignin, glycerol and ethanol) with 50 nmol **NiP**. Due to its strong light absorption properties sinapyl alcohol was studied at a lower quantity (10 mg). For the same reason lignin was also used at 0.5 mg to allow for enhanced photoabsorption by the PR system.

Photocatalysis in untreated sea water (from Gulf of Mexico, Sigma-Aldrich) with biomass-based EDs, was carried out as described above with purified water, with the difference that KP_i was replaced by untreated sea water (pH 6, 3 mL).

The capacity of the α-*cel*-CD/**NiP** system to photo-oxidize α-cellulose at different conditions was evaluated in the presence of 2.2 mg α-*cel*-CDs and 50 nmol **NiP** in various media (3 mL); KPi (pH 4.5, 6 and 8), H_2SO_4 (pH 2) and 10 M KOH.

Determination of the α-cellulose conversion yields was carried out with the α-*cel*-CD (2.2 mg)/**NiP** (50 nmol) system at various α-cellulose loadings (0.8−1.65 mg) in (KP_i pH 6, 3 mL). At 0.8 mg α-cellulose after 12, 24, and 36 h of irradiation, fresh **NiP** (50 nmol) was added to repair the photocatalytic system and maxize conversion.

Characterization of biomass oxidation products. The products of photocatalytic oxidation were determined by High Performance Liquid Chromatography Mass Spectrometry (HPLC/MS) after PR of α-cellulose, xylan, glucose and galactose with *α-cel*-CDs (2.2 mg) and **NiP** (50 nmol) in KP_i (pH 6) under full solar spectrum irradiation (AM 1.5 G, 100 mW cm^{-2}, 25 ^oC) for 6 days. Solutions from photocatalytic experiments (0.5 mL) were centrifuged at 13,000 rpm for 3 min. Next, the supernatant was separated, diluted with water (0.5 mL) and injected into a Waters XEVO G2-XS QTOF with Aquity H-Class HPLC.

The products of α-cellulose PR were also determined by NMR spectroscopy. For this purpose, uniformly labelled ^{13}C α-cellulose (10 mg) was photoreformed for 6 days under full solar spectrum irradiation (AM 1.5 G, 100 mW cm^{-2}, 25 ^oC) with *α-cel*-CDs (2.2 mg) and **NiP** (50 nmol). The spectrum was collected in D_2O.

Data treatment. All photocatalytic experiments were carried out as triplicates, and the derived performances are reported as mean values ± standard deviations (σ).

Internal Quantum Efficiency (IQE) determination. The H_2 yield was quantified upon irradiation of an O_2-free solution of the α-*cel*-CDs with monochromatic light (λ = 360 nm and light intensity (I) of 4.05 mW cm^{-2}) produced by a solar simulator (LOT LSN 254) equipped with a monochromator (LOT MSH 300). IQE was determined using equation 1 (eq. 1), where n_{H2} is the moles of photoproduced H_2, t_{irr} the irradiation time (in s), A the irradiation cross-

section (in cm^2), α the percentage of absorbed light, N_A and h are the Avogadro and Planck constants, respectively, and c the speed of light.

$$IQE\ (\%) = \frac{(2 \cdot n_{H_2} \cdot N_A \cdot h \cdot \mathrm{c})}{t_{irr} \cdot \lambda \cdot I \cdot A \cdot \alpha} \cdot 100 \qquad (\mathrm{eq.\,1})$$

Biomass conversion yield calculations. The biomass conversion yield (CY, %) was determined in KP_i pH 6 with *α-cel*-CD/**NiP** at various *α*-cellulose loadings (0.8−1.65 mg, see below **Figure S9**, **Table S11**).

CY (%) was calculated as described in equation 2 (eq. 2), where $n_{H2,\ exp}$ is the moles of H_2 produced experimentally and determined by gas chromatography as described above, and $n_{sub,\ exp}$ the moles of anhydroglucose monomer units used and determined by dividing the mass of α-cellulose (0.8−1.65 mg, **Table S11**) with the molecular weight of anhydroglucose (162.14 g mol^{-1}).

$$CY\ (\%) = \frac{n_{H_2,exp} \cdot n_{sub,ideal}}{n_{sub,exp} \cdot n_{H_2,ideal}} \cdot 100 \qquad (\mathrm{eq.\,2})$$

$n_{H2,\ ideal}\ n_{sub,\ ideal}^{-1}$ is the ideal ratio of moles H_2 to biomass substrate (anhydroglucose) as expected based on the reaction below.

$C_6H_{10}O_5 + 7H_2O \rightarrow 12\ H_2 + 6\ CO_2$

Maximum 12 equivalents of H_2 can be produced per anhydroglucose unit from α-cellulose.

Transient Absorption (TA) Spectroscopy

Ultrafast measurements. The fs-TA spectra were measured with a commercial transient absorption spectrometer (HELIOS, Ultrafast systems). The laser pulse, 800 nm, was generated from the 1 kHz Ti:sapphire regenerative amplifier (Solstice, Newport Corp) and splitted to generate both the pump and probe pulses. The pump pulse was generated by passing the 800 nm beam through a TOPAS-Prime (Light Conversion Ltd.) optical parametric amplifier to generate the 355 nm excitation light. The energy of the excitation pulse was adjusted using a natural density filter wheel. The probe light was generated by focusing the 800 nm light through a sapphire crystal (~2 mm thickness). The instrumental response time of the setup was ~200 fs. All the samples were measured with continuous stirring under argon atmosphere unless otherwise mentioned.

μs-TA measurements. Microsecond to second transient absorption measurements were acquired in the transmittance mode using an in-house developed setup as reported

previously.[2] A Nd:YAG laser (OPOTEK Opolette 355 II, 7 ns pulse width) was used as the excitation source to produce 355 nm light, which was transmitted to the sample through a light guide. The typical excitation power densities is 1 mJ cm^{-2}, with the laser repetition rate at 1.1 Hz. The probe light was generated by a 100 W Bentham IL1 quartz halogen lamp. Long pass filters (Comar Instruments) and IR filters (H_2O, 5 cm path length) were positioned between the halogen lamp and the sample to minimize short wavelength irradiation and the heating. The transmitted light from the sample was collected and relayed to a monochromator (Oriel Cornerstone 130) through a long pass filter to select the specific probe wavelength. Acquisitions were triggered by a photodiode (Thorlabs DET10A) exposed to laser scatter. Time-resolved intensity of the transmitted light were collected with a Si photodiode (Hamamatsu S3071). Data at times faster than 3.6 ms were amplified by customized electronics (Costronics) and recorded by an oscilloscope (Tektronics DPO3012), whereas data slower than 3.6 ms were simultaneously recorded on a National Instrument DAQ card (NI USB-6251). Kinetic traces were typically obtained from an average of 64 laser pulses. Unless otherwise stated, the samples were measured under similar conditions with photocatalytic measurements, using 2.2 mg of α-*cel*-CDs in KPi buffer solution (pH 6), upon the addition of **NiP** (50 nmol) and/or EDTA (0.1 M, pH 6) under Ar.

Table S1. Photo-generated H_2 in purified water with EDTA using CDs from α-cellulose (α-*cel*-CDs) synthesized at 320 ºC for 100 h.[a]

Entry	Precursor	CDs / mg	t_{irr} / h	H_2 / µmol	± σ / µmol	TON_{NiP} / mol H_2 (mol cat)$^{-1}$	± σ / mol H_2 (mol cat)$^{-1}$	Activity/ µmol H_2 (g CDs)$^{-1}$ h^{-1}	± σ / µmol H_2 (g CDs)$^{-1}$ h^{-1}
1	α-cellulose	0.03	6	0.9	0.04	18.7	0.8	13450.0	540.3
2			24	1.2	0.03	24.0	0.7	-	-
3	α-cellulose	0.06	6	1.7	0.02	34.8	0.4	11685.8	586.2
4			24	2.2	0.03	44.8	0.6	-	-
5	α-cellulose	0.10	6	3.4	0.10	67.5	2.1	10109.3	339.6
6			24	4.1	0.20	81.9	4.4	-	-
7	α-cellulose	0.30	6	5.4	0.05	107.9	0.9	7146.7	304.8
8			24	6.3	0.40	126.6	7.3	-	-
9	α-cellulose	1.0	6	10.5	0.20	209.1	4.4	4326.6	141.0
10			24	10.9	0.20	219.0	3.7	-	-
11	α-cellulose	2.20	6	11.0	0.50	220.1	9.8	1101.1	36.2
12			24	15.6	0.70	312.8	14.6	-	-
13	α-cellulose	2.80	6	13.4	0.40	268.2	8.8	1410.4	82.1
14			24	15.1	0.70	301.7	13.9	-	-

[a]Irradiation (AM 1.5G, 100 mW cm^{-2}) for 6 and 24 h, 25 °C, in the presence of **NiP** (50 nmol), EDTA (0.1 M, pH 6, 3 mL).

Table S2. Photo-generated H_2 in purified water with EDTA using *g*-N-CDs from aspartic acid synthesized at 320 ºC for 100 h.[a]

Entry	Precursor	CDs / mg	t_{irr} / h	H_2 / μmol	± σ / μmol	TON_{NiP} / mol H_2 (mol cat)$^{-1}$	± σ / mol H_2 (mol cat)$^{-1}$	Activity/ μmol H_2 (g CDs)$^{-1}$ h^{-1}	± σ / μmol H_2 (g CDs)$^{-1}$ h^{-1}
1	aspartic acid	0.03	4	0.4	0.0	41.4	2.5	7424.5	1164.0
2	aspartic acid	0.06	4	0.8	0.1	76.9	2.5	6953.0	1082.9
3	aspartic acid	0.10	4	1.3	0.1	131.8	2.5	6553.0	768.2
4	aspartic acid	0.30	4	2.0	0.1	200.0	6.6	3991.9	208.8
5	aspartic acid	1.00	4	2.4	0.1	239.2	6.8	1141.9	75.9
6	aspartic acid	2.20	4	5.2	0.3	520.0	13.0	724.6	56.2
7	aspartic acid	2.80	4	5.8	0.3	580.1	12.0	585.6	49.3

[a]Irradiation (AM 1.5G, 100 mW cm^{-2}) for 4 h, 25 °C, **NiP** (10 nmol), EDTA (0.1 M, pH 6, 3 mL). The systems do not produce additional H_2 at longer irradiation times.

Table S3. Photo-H_2 production in purified water with EDTA using *g*-CDs from citric acid synthesized via a two-step process.[a, b]

Entry	Precursor	CDs / mg	t_{irr} / h	H_2 / μmol	± σ / μmol	TON_{NiP} / mol H_2 (mol cat)$^{-1}$	± σ / mol H_2 (mol cat)$^{-1}$	Activity/ μmol H_2 (g CDs)$^{-1}$ h^{-1}	± σ / μmol H_2 (g CDs)$^{-1}$ h^{-1}
1	citric acid	0.03	4	0.1	0.0	6.6	2.9	1141.9	75.9
2	citric acid	0.06	4	0.1	0.0	12.5	2.5	1074.5	194.8
3	citric acid	0.10	4	0.2	0.0	20.4	2.9	859.6	111.3
4	citric acid	0.30	4	0.4	0.1	44.3	5.5	562.9	92.4
5	citric acid	1.00	4	0.5	0.1	53.2	8.8	132.9	22.0
6	citric acid	2.20	4	1.3	0.1	130.9	6.5	157.1	7.9
7	citric acid	2.80		1.4	0.1	138.1	6.9	132.9	6.6

[a]Irradiation (AM 1.5G, 100 mW cm^{-2}) for 4 h, 25 °C, in the presence of **NiP** (10 nmol), EDTA (0.1 M, pH 6, 3 mL). The systems do not produce additional H_2 at longer irradiation times; [b]CDs were synthesized via a two-step thermal process; citric acid was calcinated at 180 ºC for 40 h and then at 320 ºC for 100 h.

Table S4. Photo-H_2 production in purified water with EDTA using *a*-CDs from citric acid synthesized at 180 ºC for 40 h.[a]

Entry	Precursor	CDs / mg	t_{irr} / h	H_2 / μmol	± σ / μmol	TON_{NiP} / mol H_2 (mol cat)$^{-1}$	± σ / mol H_2 (mol cat)$^{-1}$	Activity/ μmol H_2 (g CDs)$^{-1}$ h^{-1}	± σ / μmol H_2 (g CDs)$^{-1}$ h^{-1}
1	citric acid	0.03	4	0.0	0.0	0.3	0.6	0.0	0.0
2	citric acid	0.06	4	0.0	0.0	0.0	0.0	0.0	0.0
3	citric acid	0.10	4	0.0	0.0	2.9	0.8	166.9	41.8
4	citric acid	0.30	4	0.1	0.0	5.2	0.6	132.9	24.8
5	citric acid	1.00	4	0.3	0.0	24.9	2.9	157.6	19.9
6	citric acid	2.20	4	0.3	0.0	32.5	1.6	71.6	3.6
7	citric acid	2.80	4	0.7	0.0	74.0	3.7	131.3	6.6

[a]Irradiation (AM 1.5G, 100 mW cm^{-2}) for 4 h, 25 °C, in the presence of **NiP** (10 nmol), EDTA (0.1 M, pH 6, 3 mL). The systems do not produce additional H_2 at longer irradiation times.

Table S5. Photocatalytic performances of metal-free carbonaceous photoabsorbers in purified water.[a]

Entry	Photoabsorbers	Catalyst	ED	H_2 / µmol	Activity / µmol g^{-1} h^{-1}	TON_{cat}	QE[b] / %	Ref.
1	*a*-CD	**NiP**	EDTA	0.6	397	64	1.4	[S3]
2	*a*-CD	**NiP**	TCEP[c]/AA[d]	10.9	53	1094	n.d.	[S5]
3	*g-CD*	**NiP**	EDTA	0.5	1,549	45	n.d.	[S2]
4	*g-N*-CD	**NiP**	EDTA	2.8	7,950	277	5.2	[S2]
5	CD (yeast)	Pt	TEOA[e]	n.d	31	n.d.	n.d.	[S6]
6	$^{NH2}CN_x$	**NiP**	EDTA	3.3	437	166	0.4	[S7]
7	$^{NCN}CN_x$	**NiP**	4-MBA[f]	21.3	311	425	15.2	[S8]
8	Ultr. $^{NCN}CN_x$[k]	**NiP**[g]	4-MBA	31.7	39,310	79.2	22	[S9]
9	Ultr. $^{NCN}CN_x$	**NiP**[h]	4-MBA	9.23	5,620	193	22	[S9]
8	*g*-C_3N_4	Pt	TEOA	n.d.	20,000	641	26.5	[S10]

[a]These systems use a sacrificial ED and non-precious (**NiP**) as well as noble-metal catalysts (Pt) in purified water; [b]QE: quantum efficiency; [c]TCEP: tris(carboxyethyl)phosphine; [d]AA: ascorbic acid; [e]TEOA: triethanolamine; [f]4-MBA: 4-methylbenzyl alcohol [g]**NiP**: 400 nmol; [h]**NiP**: 50 nmol; [k]Ultr. ^{NCN}CNx: 0.5 mg.

Table S6. Photocatalytic H_2 production in purified water with α-*cel*-CDs using different biomass compounds and 4-MBA[a] as substrates.[b]

Entry	Substrate	t_{irr} / h	H_2 / μmol	± σ / μmol	TON cat / mol H_2 (mol cat)$^{-1}$	± σ / mol H_2 (mol cat)$^{-1}$	Activity/ μmol H_2 (g CDs)$^{-1}$ h^{-1}	± σ / μmol H_2 (g CDs)$^{-1}$ h^{-1}
1	4-MBA[b]	6	3.7	0.2	73.3	3.7	572.0	28.6
		24	3.8	0.2	75.7	3.8	-	-
2	α-cellulose	24	5.0	0.2	101.0	3.4	268.2	4.5
3	cellobiose	24	6.7	0.4	134.7	7.4	509.7	8.2
4	glucose	24	6.1	0.2	121.6	3.4	413.1	73.0
5	xylan	24	3.6	0.3	71.3	6.6	20.1	1.4
6	xylose	24	6.1	0.2	122.2	3.9	435.0	25.6
7	galactose	24	8.8	0.2	176.7	3.5	455.8	38.7
8	lignin[c]	24	0.03	0.002	0.60	0.03	14.0	0.7
9	lignin[d]	24	7.8	0.5	155.7	10.39	324.3	6.4
10	sinapyl alcohol[e]	24	0.03	0.002	0.60	0.03	14.0	0.7
11	glycerol	24	8.5	0.1	170.3	1.4	495.6	23.4
12	ethanol	i24	6.7	1.0	135.0	20.8	458.3	35.5

[a]4-MBA: 4-methylbenzyl alcohol; [b]Irradiation (AM 1.5G, 100 mW cm^{-2}) for 24 h, 25 °C, in the presence of **NiP** (50 nmol), in KP_i (pH 6, 3 mL); [c]100 mg lignin; [d]0.5 mg lignin; [e]10 mg sinapyl alcohol.

Table S7. Control photocatalytic experiments with and without α-*cel*-CDs, EDTA or α-cellulose as ED and **NiP**.[a]

Entry	Reaction medium	CDs / mg	ED / 0.1 M	NiP / nmol	H_2 / μmol	± σ / μmol	Activity/ μmol H_2 (g CDs)$^{-1}$ h^{-1}	± σ / μmol H_2 (g CDs)$^{-1}$ h^{-1}
**No UV cut-off filter (λ > 300 nm)**								
1	Purified water	2.2	-	50	0.1	0.01	19.0	2.8
2	Purified water	-	EDTA	50	-	-	-	-
3	Purified water	-	α-cellulose	50	-	-	-	-
4	Purified water	2.2	EDTA	-	-	-	-	-
5	Purified water	2.2	α-cellulose	-				
6	Sea water	2.2	EDTA	-	-	-	-	-
7	Sea water	2.2	-	50	5.6	0.4	293.6	42.8
**UV cut-off filter (λ > 400 nm)**								
8	Purified water	2.2	EDTA	50	4.4	0.4	233.8	11.3
9	Purified water	2.2	α-cellulose	50	0.2	0.01	3.6	0.2

[a]These experiments were carried out both in purified and untreated sea water, under AM 1.5 G (100 mW cm^{-2}) irradiation at 25 ºC. The results in the presence of an optical filter (λ > 400 nm) with all system components are also provided, to indicate the activity of the α-*cel*-CDs as visible-light photoabsorbers.

Table S8. Photocatalytic H_2 production in purified water using *g*-N-CDs with different biomass substrates.[a]

Entry	Substrates	t_{irr} / h	H_2 / µmol	± σ / µmol	TON cat / mol H_2 (mol cat)$^{-1}$	± σ / mol H_2 (mol cat)$^{-1}$	Activity/ µmol H_2 (g CDs)$^{-1}$ h^{-1}	± σ / µmol H_2 (g CDs)$^{-1}$ h^{-1}
1	α-cellulose	24	4.2	0.5	83.3	9.9	728.2	176.2
2	cellobiose	24	5.3	0.6	105.7	11.3	1988.2	158.0
3	glucose	24	4.9	0.7	97.1	14.7	1969.0	180.9
4	xylan	24	0.7	0.04	13.7	0.7	32.2	1.6
5	xylose	24	4.9	0.03	97.3	0.70	1942.7	292.4
6	galactose	24	5.9	0.3	117.2	5.1	2001.4	393.4
7	lignin[b]	24	0.03	0.002	0.60	0.03	60.0	3.0
8	lignin[c]	24	3.8	1.3	76.7	26.1	1431.1	402.5
9	sinapyl alcohol[d]	24	0.0	0.0	0.0	0.0	0.0	0.0
10	glycerol	24	3.1	0.2	62.6	3.3	1371.3	46.4
11	ethanol	24	3.9	0.6	77.6	11.1	1576.7	82.3

[a]Irradiation (AM 1.5G, 100 mW cm^{-2}) for 24 h, 25 °C, in the presence of **NiP** (50 nmol), KP_i (pH 6, 3 mL); [b]100 mg lignin; [c]0.5 mg lignin; [d]10 mg sinapyl alcohol

Table S9. Photocatalytic H_2 production in purified water using *a*-CDs with different biomass substrates.[a]

Entry	Substrates	t_{irr} / h	H_2 / μmol	± σ / μmol	TON_{NiP} / mol H_2 (mol cat)$^{-1}$	± σ / mol H_2 (mol cat)$^{-1}$	Activity/ μmol H_2 (g CDs)$^{-1}$ h^{-1}	± σ / μmol H_2 (g CDs)$^{-1}$ h^{-1}
1	α-cellulose	24	0.5	0.1	10.6	2.5	3.8	0.9
2	cellobiose	24	0.7	0.1	14.2	2.2	11.5	0.9
3	glucose	24	0.6	0.04	12.6	0.8	10.9	0.8
4	xylan	24	0.08	0.003	1.6	0.05	0.3	0.01
5	xylose	24	0.6	0.1	12.8	1.9	7.9	2.5
6	galactose	24	0.7	0.01	14.4	0.3	11.6	0.5
7	lignin[b]	24	0.0	0.0	0.0	0.0	0.0	0.0
9	sinapyl alcohol[c]	24	0.0	0.0	0.0	0.0	0.0	0.0
10	glycerol	24	1.7	0.1	34.4	1.7	20.0	1.0
11	ethanol	24	0.6	0.03	12.2	0.6	7.2	0.4

[a]Irradiation (AM 1.5G, 100 mW cm^{-2}), 25 °C, in the presence of **NiP** (50 nmol), KP_i (pH 6, 3 mL); [b]100 mg lignin; [c]10 mg sinapyl alcohol.

Table S10. PR of α-cellulose into H_2 with the α-*cel*-CD/**NiP** system under different conditions.[a]

Entry	Conditions	t_{irr} / h	H_2 / µmol	± σ / µmol	TON_{NiP} / mol H_2 (mol cat)$^{-1}$	± σ / mol H_2 (mol cat)$^{-1}$	Activity/ µmol H_2 (g CDs)$^{-1}$ h^{-1}	± σ / µmol H_2 (g CDs)$^{-1}$ h^{-1}
1	H_2SO_4 (pH 2)	24	1.2	0.1	24.7	1.2	60.0	3.0
2	KPi (pH 4.5)	24	3.1	0.2	61.8	3.1	168.0	8.4
3	KPi (pH 6)	24	5.0	0.2	101.0	3.4	268.2	4.5
4	KPi (pH 8)	24	3.6	0.2	72.9	3.6	514.0	25.7
5	10 M KOH	24	0.0	0.0	0.0	0.0	0.0	0.0

[a]Irradiation (AM 1.5G, 100 mW cm^{-2}) for 24 h, 25 °C, using **NiP** as co-catalyst (50 nmol).

Table S11. α-cellulose conversion yields with the α-*cel*-CD/**NiP** system.[a]

Entry	α-cellulose / mg	t_{irr} / h	H_2 / µmol	± σ / µmol	Anhydroglucose / µmol[b]	Theoretical H_2 / µmol[c]	Conversion Yield / %
1	1.65	12	7.2	0.4	10.2	122.1	5.9
2	1.18	12	7.3	0.4	7.3	87.3	8.4
3	0.81[d]	12[d]	8.0	0.9	5.0	59.9	13.4
4		24	15.1	0.1	5.0	59.9	25.2
5		36	19.1	0.7	5.0	59.9	31.9
6		48	20.4	0.7	5.0	59.9	34.1

[a]Irradiation (AM 1.5G, 100 mW cm^{-2}), 25 ºC, at optimum conditions (KP_i pH 6, 50 nmol **NiP**) upon varying the α-cellulose loading (0.81−1.65 mg); [b]The moles of anhydroglucose units that are available for PR at different α-cellulose loadings, were calculated by dividing the respective masses used by the molecular weight of anhydroglucose (162.14 g mol^{-1}); [c]The theoretical moles of H_2 expected upon full conversion of the amount of α-cellulose used in each case, was calculated by multiplying the amount of anhydroglucose units by 12. This number represents the equivalents of H_2 which are produced per anhydroglucose unit[11]; [d]At 0.81 mg of α-cellulose after 12, 24, and 36 h of irradiation, fresh **NiP** (50 nmol) was added to overcome the co-catalyst degradation effect and test the maximum performance of the system.

Table S12. Photocatalytic H_2 production under real-world conditions using sea water, *α-cel*-CDs and different biomass substrates.[a]

Entry	Substrate	t_{irr} / h	H_2 / µmol	± σ / µmol	TON cat / mol H_2 (mol cat)$^{-1}$	± σ / mol H_2 (mol cat)$^{-1}$	Activity/ µmol H_2 (g CDs)$^{-1}$ h^{-1}	± σ / µmol H_2 (g CDs)$^{-1}$ h^{-1}
1	α-cellulose	24	2.7	0.2	53.0	4.5	42.2	14.8
2	cellobiose	24	7.2	0.9	143.8	18.3	115.8	47.3
3	glucose	24	7.7	0.3	153.2	6.5	170.0	4.2
4	xylan	24	0.7	0.1	13.6	2.1	13.2	2.1
5	xylose	24	8.0	0.01	159.4	0.2	156.4	3.2
6	galactose	24	8.4	0.1	168.1	0.1	135.6	1.1
7	lignin[b]	24	0.03	0.002	0.60	0.03	14.0	0.7
9	sinapyl alcohol[c]	24	0.05	0.01	0.90	0.20	0.9	0.2
10	glycerol	24	7.2	0.5	143.8	10.5	144.7	8.3
11	ethanol	24	6.2	0.2	123.6	3.2	118.0	3.3

[a]Irradiation (AM 1.5G, 100 mW cm^{-2}) for 24 h, 25 °C, in the presence of **NiP** (50 nmol), sea water (pH 6, 3 mL); [b]100 mg lignin; [c]10 mg sinapyl alcohol.

Table S13. Photocatalytic H_2 production under real-world conditions using sea water, *g*-N-CDs and different biomass substrates.[a]

Entry	Substrate	t_{irr} / h	H_2 / µmol	± σ / µmol	TON cat / mol H_2 (mol cat)$^{-1}$	± σ / mol H_2 (mol cat)$^{-1}$	Activity/ µmol H_2 (g CDs)$^{-1}$ h^{-1}	± σ / µmol H_2 (g CDs)$^{-1}$ h^{-1}
1	α-cellulose	24	0.6	0.2	11.7	1.6	33.8	8.1
2	cellobiose	24	2.2	0.1	43.3	2.4	372.3	17.5
3	glucose	24	2.3	0.1	46.2	1.6	401.6	47.5
4	xylan	24	0.2	0.01	4.6	0.2	11.8	1.7
5	xylose	24	1.8	0.2	36.1	3.3	338.4	71.2
6	galactose	24	0.9	0.1	17.9	1.9	110.8	23.0
7	lignin[b]	24	0.0	0.0	0.0	0.0	0.0	0.0
9	sinapyl alcohol[c]	24	0.0	0.0	0.0	0.0	0.0	0.0
10	glycerol	24	0.9	0.03	18.3	0.7	118.7	4.5
11	ethanol	24	0.8	0.04	15.4	0.8	128.3	6.7

[a]Irradiation (AM 1.5G, 100 mW cm^{-2}) for 24 h, 25 °C, in the presence of **NiP** (50 nmol), sea water (pH 6, 3 mL); [b]100 mg lignin; [c]10 mg sinapyl alcohol.

Table S14. Photocatalytic H_2 production under real-world conditions using sea water, *a*-CDs and different biomass substrates.[a]

Entry	Substrate	t_{irr} / h	H_2 / μmol	± σ / μmol	TON cat / mol H_2 (mol cat)$^{-1}$	± σ / mol H_2 (mol cat)$^{-1}$	Activity/ μmol H_2 (g CDs)$^{-1}$ h^{-1}	± σ / μmol H_2 (g CDs)$^{-1}$ h^{-1}
1	α-cellulose	24	0.04	0.002	0.80	0.04	0.2	0.01
2	cellobiose	24	0.10	0.005	2.1	0.1	0.9	0.04
3	glucose	24	0.08	0.004	1.7	0.09	0.4	0.01
4	xylan	24	0.03	0.001	0.5	0.03	0.1	0.01
5	xylose	24	0.20	0.01	4.0	0.20	2.6	0.10
6	galactose	24	0.30	0.01	5.9	0.30	2.4	0.10
7	lignin[b]	24	0.00	0.00	0.0	0.00	0.0	0.00
9	sinapyl alcohol[c]	24	0.03	0.002	0.6	0.03	0.1	0.01
10	glycerol	24	0.15	0.008	3.0	0.2	1.3	0.06
11	ethanol	24	0.30	0.016	6.5	0.3	2.5	0.13

[a]Irradiation (AM 1.5G, 100 mW cm^{-2}) for 24 h, 25 °C, in the presence of **NiP** (50 nmol), sea water (pH 6, 3 mL); [b]100 mg lignin; [c]10 mg sinapyl alcohol.

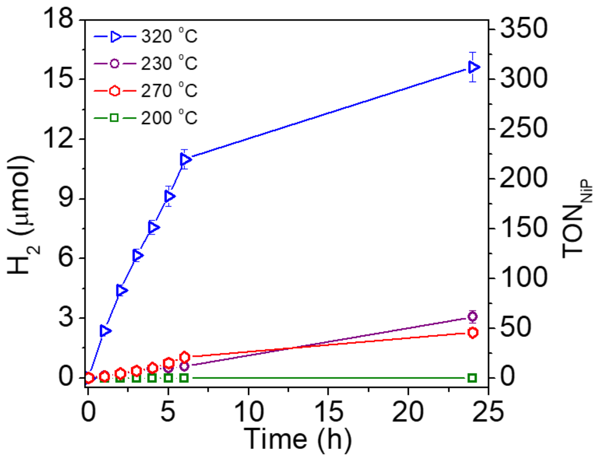

Figure S1. Photocatalytic H_2 production from *α-cel*-CDs (2.2 mg) synthesized upon calcination of *α*-cellulose at 200, 230, 270 and 320 ºC. Conditions: all experiments were carried out under irradiation with simulated solar light (AM 1.5 G, 100 mW cm^{-2}) in the presence of **NiP** (50 nmol) and EDTA (0.1 M, pH 6, 3 mL) under N_2 atmosphere containing 2% CH_4 at 25 ºC.

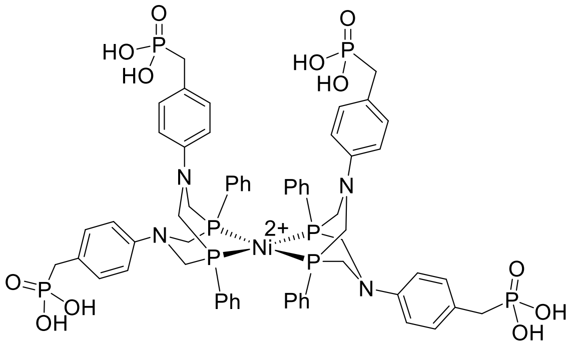

Figure S2. Chemical structure of the Ni bis(diphosphine) (**NiP**) H_2 evolution catalyst (bromide counter ions not shown).

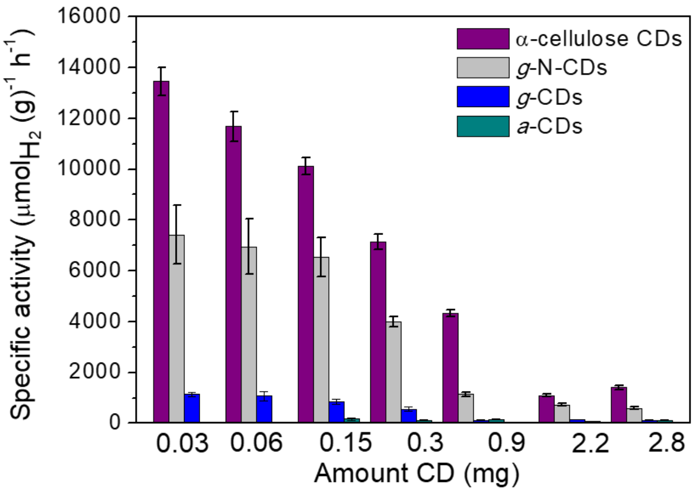

Figure S3. Specific activities using *α-cel*-CDs, *g*-N-CDs, *g*-CDs and *a*-CDs as photoabsorbers at various quantities (0.03−2.8 mg) in the presence of 50 nmol **NiP**. The photocatalytic experiments were carried out in aqueous EDTA solutions (0.1 M, pH 6, 3 mL) for 24 h under simulated sunlight irradiation (AM 1.5 G, 100 mW cm^{-2}) at 25 ºC.

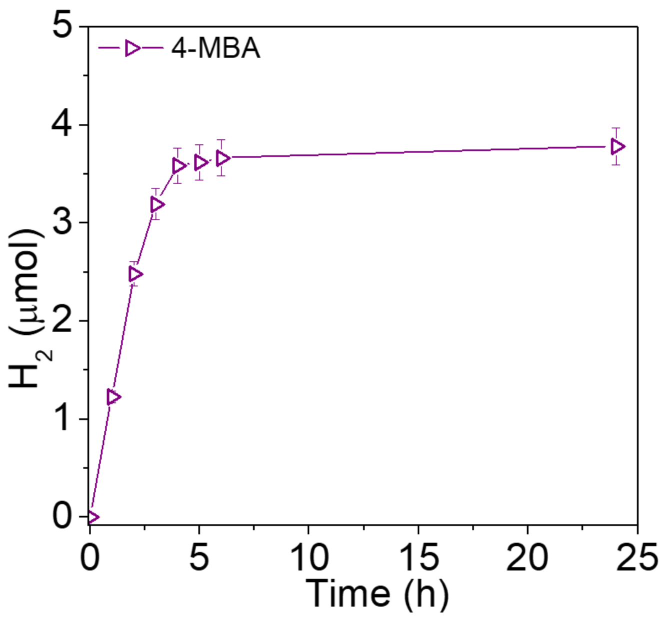

Figure S4. Photocatalytic experiments using α-*cel*-CDs as photoabsorbers with **NiP** (50 nmol) and 4-MBA (30 μmol) as sacrificial ED in KP_i (pH 6, 3 mL) under simulated sunlight irradiation (AM 1.5 G, 100 mW cm^{-2}) at 25 ºC.

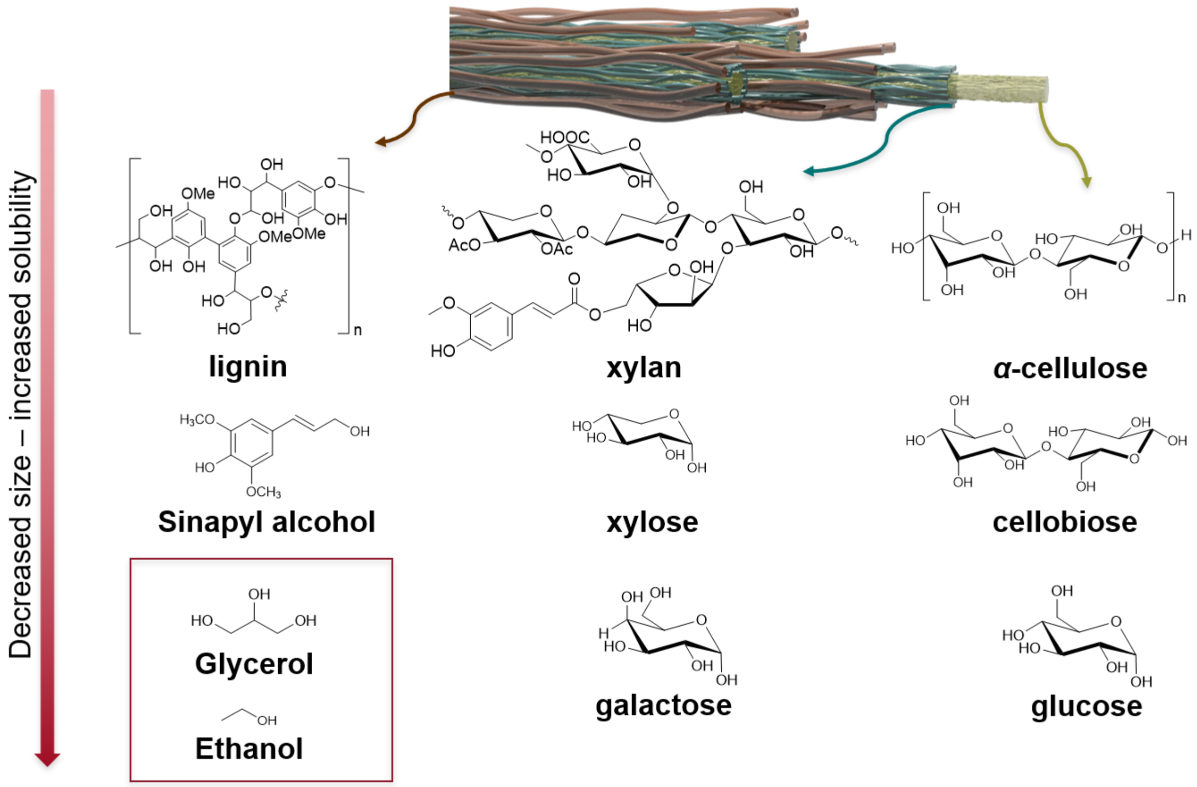

Figure S5. Chemical structures of the biomass and biomass-derived substrates studied as EDs in this study.

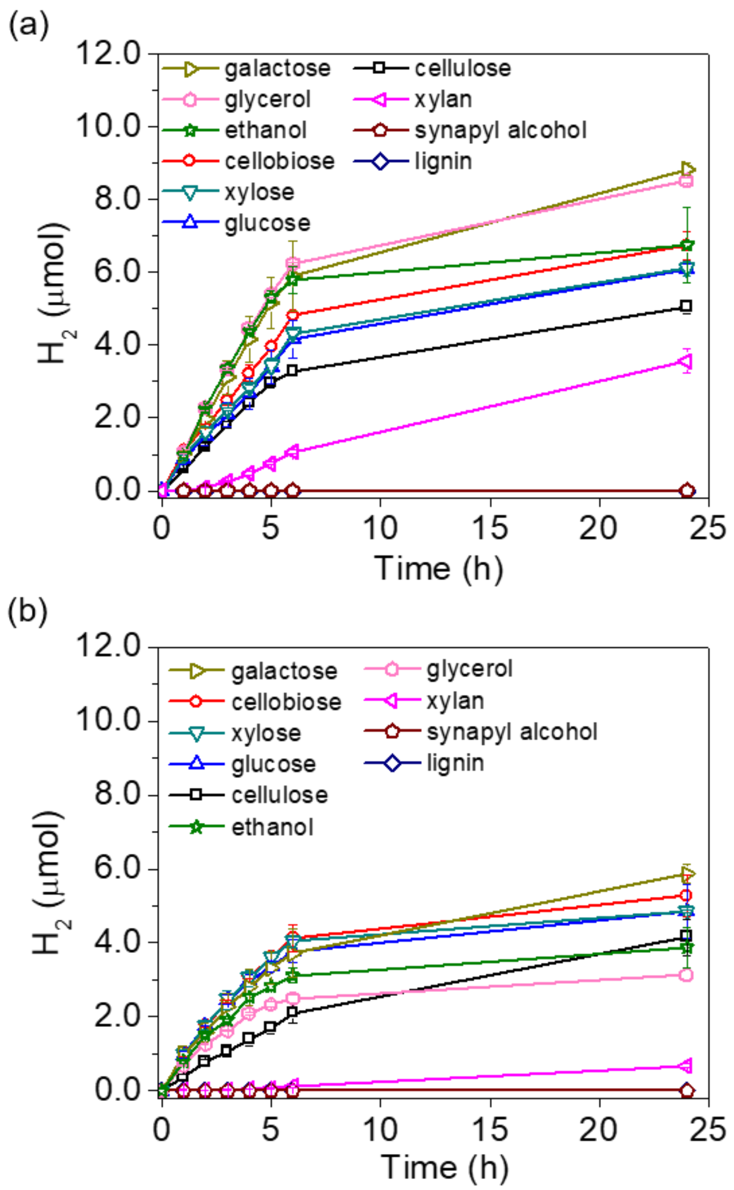

Figure S6. Photocatalytic H_2 production with (a) α-*cel*-CDs and (b) *g*-N-CDs in purified water. Pure lignocellulosic components (100 mg) and biomass-derived chemicals (100 mg) were employed as EDs. All experiments were carried out under simulated sunlight irradiation (AM 1.5 G, 100 mW cm^{-2}) for 24 h at 25 ºC in the presence of **NiP** (50 nmol).

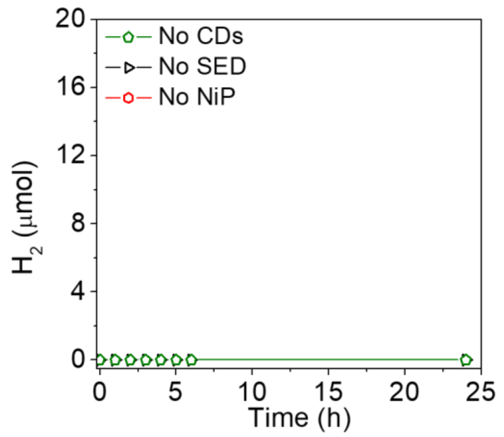

Figure S7. Control photocatalytic experiments carried out under simulated sunlight irradiation (AM 1.5 G, 100 mW cm^{-2}) at 25 ºC in the absence of α-*cel*-CDs, **NiP** or EDTA.

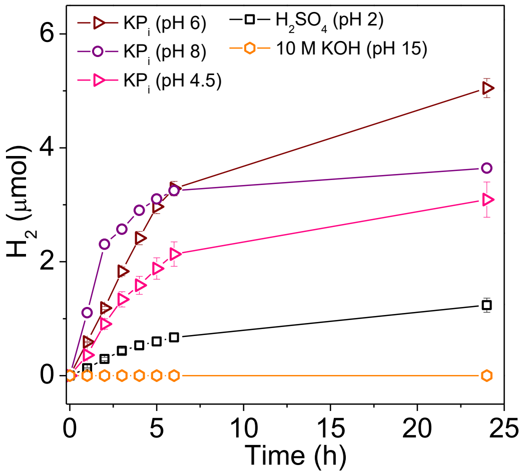

Figure S8. Photocatalytic H_2 production using α-*cel*-CDs (2.2 mg) as photoabsorbers, **NiP** as the hydrogen evolution cocatalyst and α-cellulose as ED (100 mg) in different media; KP_i (pH 4.5, 6 and 8), H_2SO_4 (pH 2) and 10 M KOH (~pH 15). All experiments were carried out under simulated sunlight irradiation (AM 1.5 G, 100 mW cm^{-2},) at 25 ºC.

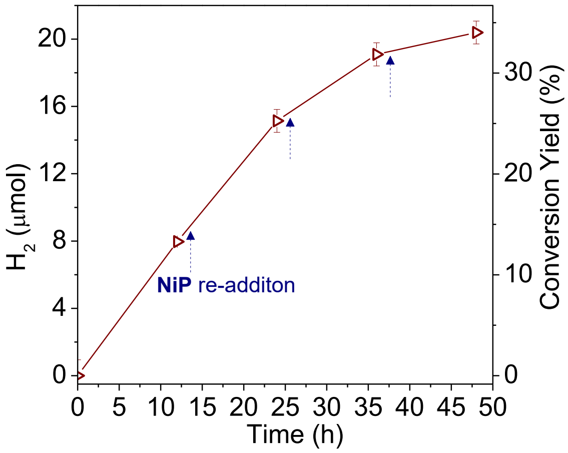

Figure S9. Photocatalytic H_2 production using α-*cel*-CDs (2.2 mg) and α-cellulose as ED (0.81 mg) in aqueous KP_i (pH 6, 3 mL) under simulated solar irradiation (AM 1.5 G, 100 mW cm^{-2}) at 25 ºC with several readditions of **NiP** (50 nmol) as indicated by the blue arrows to improve the stability of the PR system.

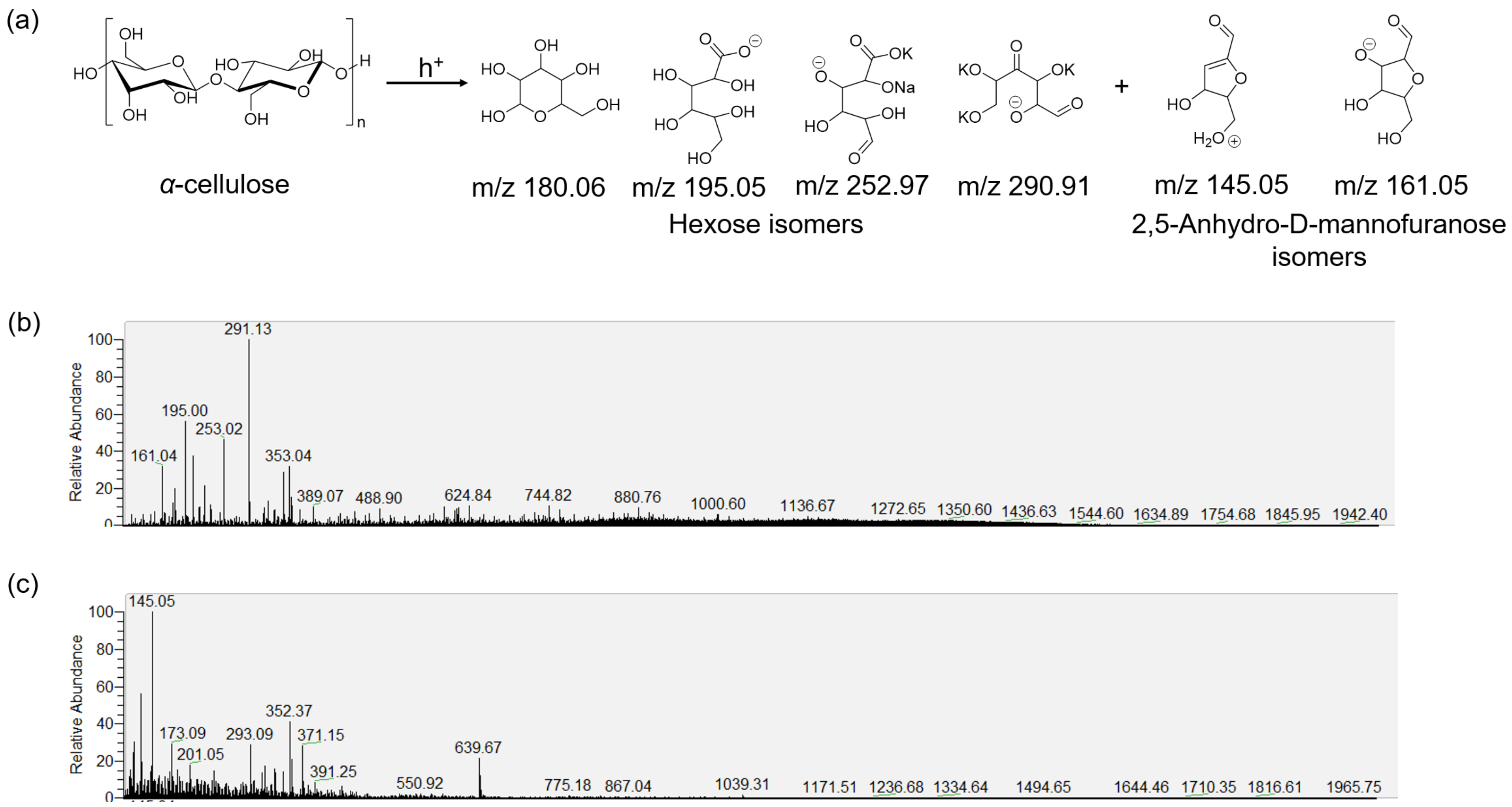

Figure S10. (a) Proposed structures of hexose and 2,5-anhydro-D-mannofuranose isomers produced upon PR of *α*-cellulose with *α-cel*-CDs (2.2 mg) and **NiP** (50 nmol) in KP_i (pH 6) under simulated sunlight irradiation (AM 1.5 G, 100 mW cm^{-2}) at 25 ºC for 6 days, as derived from HPLC/MS analysis in (b) negative and (c) positive ionization modes.

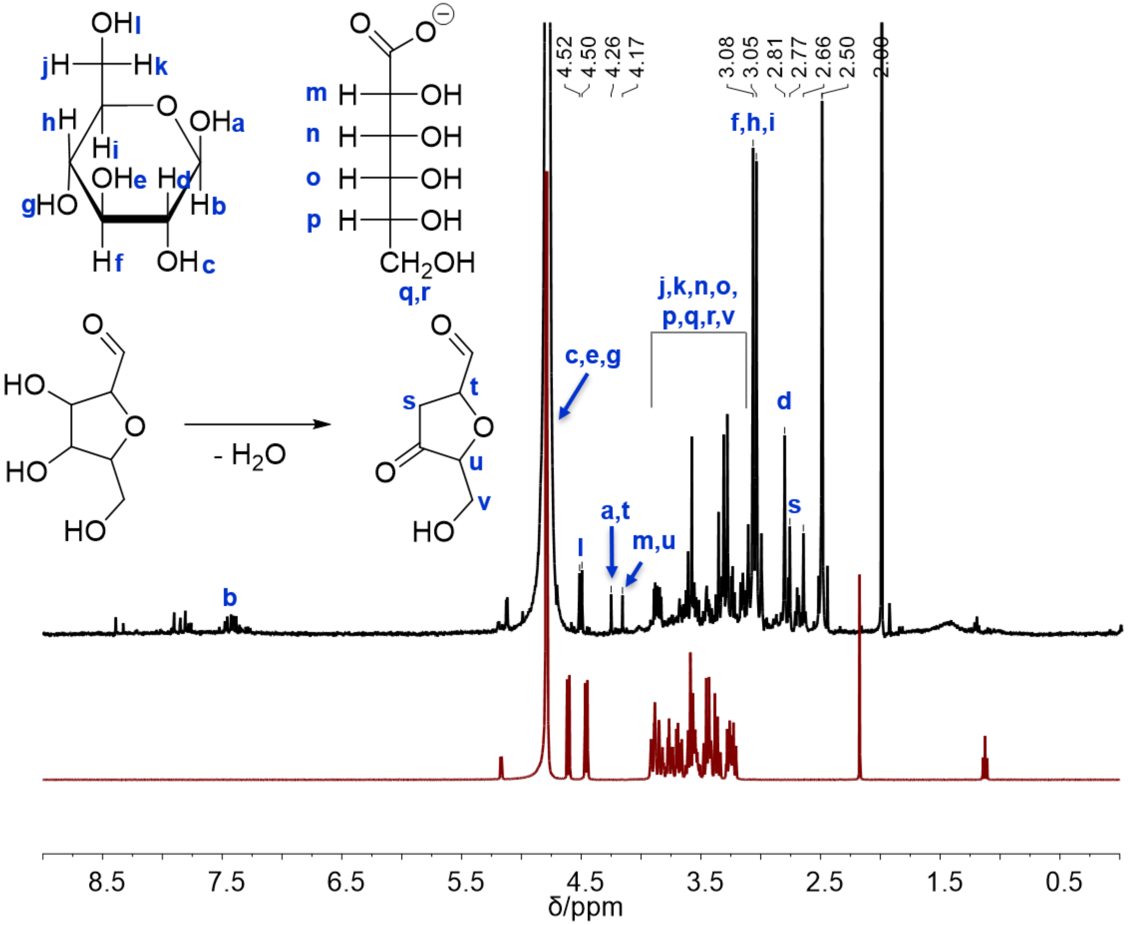

Figure S11. ^{1}H NMR spectrum in D_2O recorded after PR of *α*-cellulose (black) with *α-cel*-CDs (2.2 mg) and **NiP** (50 nmol) in KP_i (pH 6) under simulated sunlight irradiation (AM 1.5 G, 100 mW cm^{-2}) at 25 ºC. For comparison, the ^{1}H NMR spectrum of *α*-cellulose before PR is shown in red. The chemical structures of the main $C_6H_{12}O_6$ (δ = 2.81, 3.05, 3.08, 3.2-3.9, 4.17, 4.26, 4.52 and 7.5 ppm) and $C_6H_{10}O_5$ oxidation products (2,5-anhydro-D-mannofuranose isomers; δ = 2.66, 2.77, 3.80, 4.17, 4.26 ppm), along with their characteristic chemical shifts are also displayed.

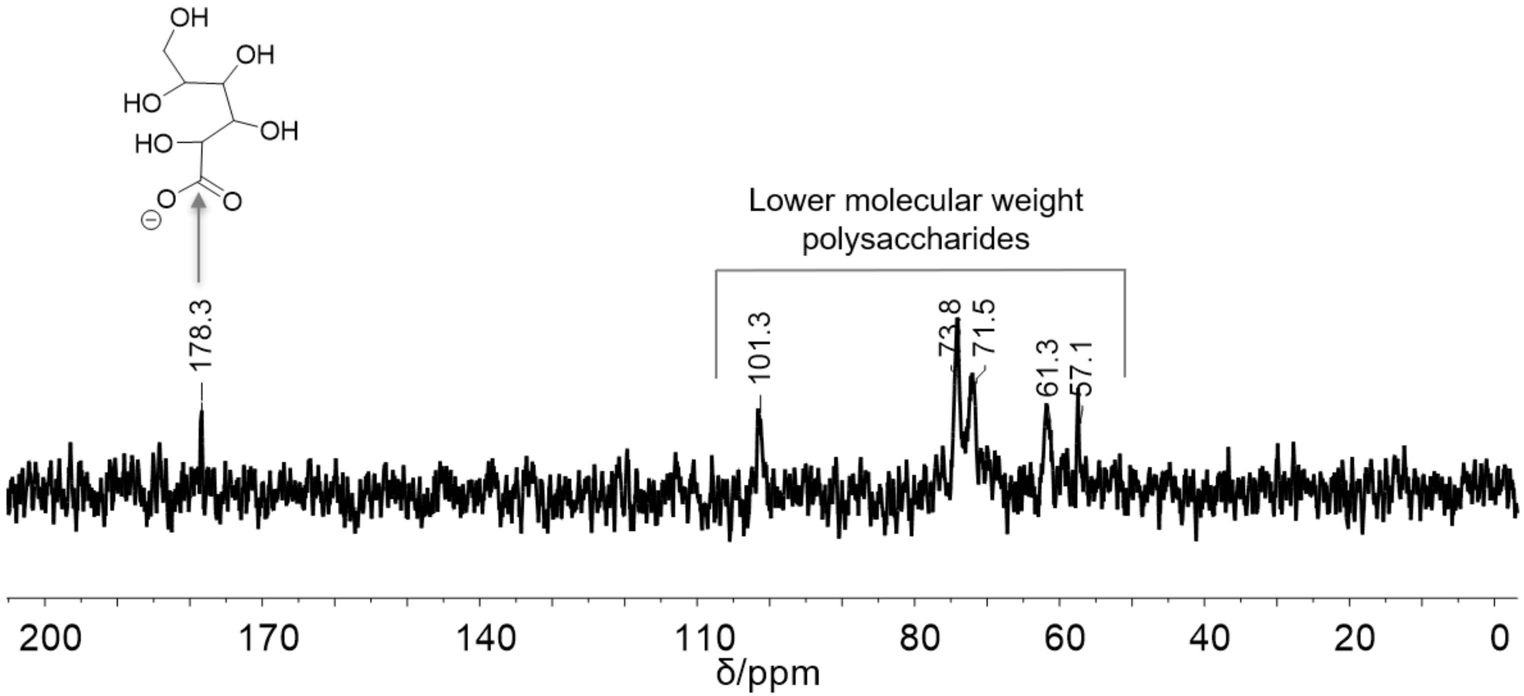

Figure S12. ^{13}C NMR spectrum (in D_2O) recorded after PR of uniformly labelled ^{13}C α-cellulose (10 mg) for 6 days under simulated sunlight irradiation (AM 1.5 G, 100 mW cm^{-2}) at 25 ºC with *α-cel*-CDs (2.2 mg) and **NiP** (50 nmol). The chemical shifts of the main PR product 2,3,4,5,6-pentahydroxyhexanoate (carboxyl carbon, δ = 178.3 ppm) along with lower molecular weight polysaccharides (δ = 50−100 ppm) are shown.

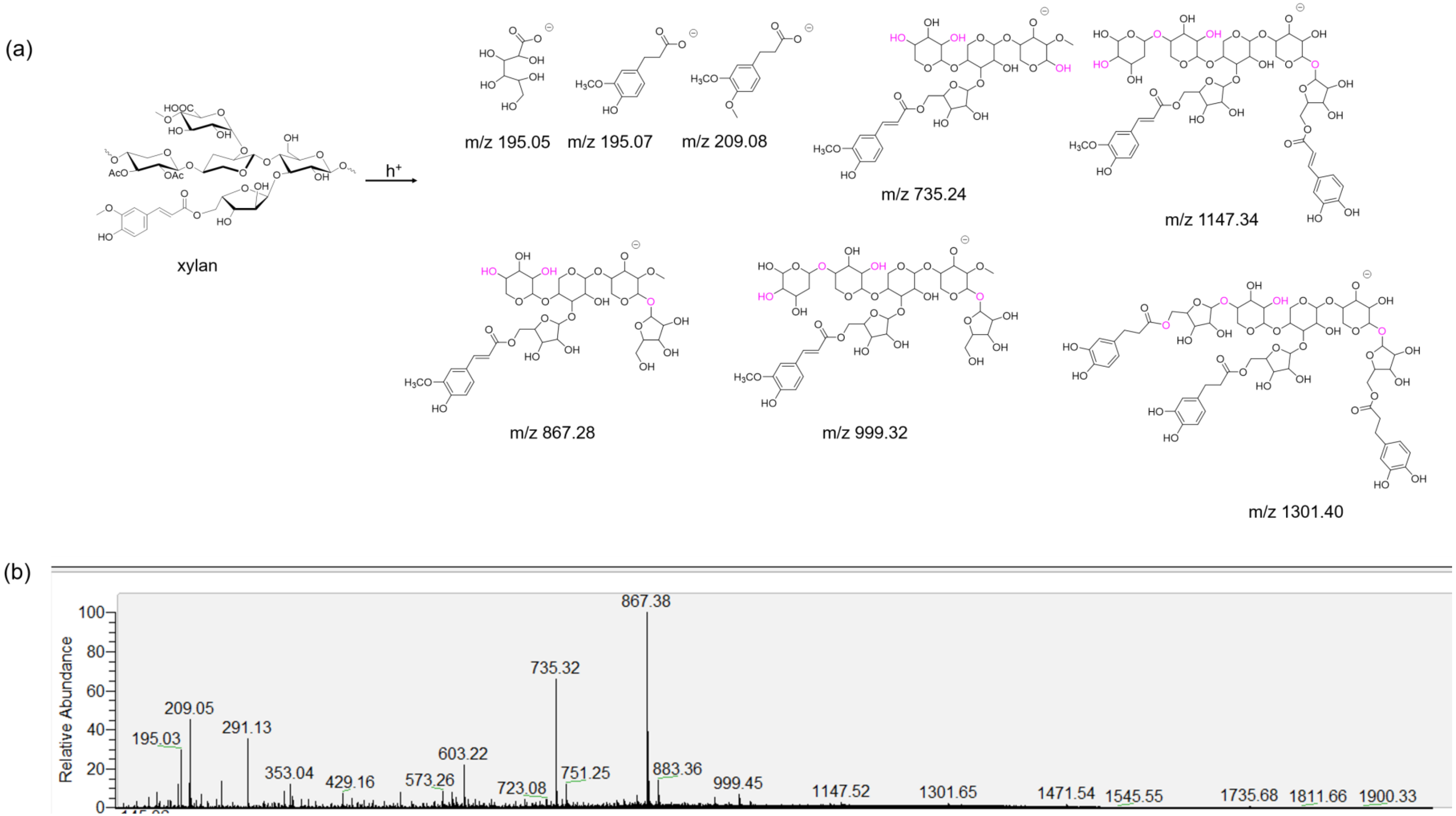

Figure S13. (a) Proposed structures of hydroferulic acid and its isomers as well as more complex xylan depolymerization products derived from PR of xylan with *α-cel*-CDs (2.2 mg) and **NiP** (50 nmol) in KP_i (pH 6) under simulated sunlight irradiation (AM 1.5 G, 100 mW cm^{-2}) at 25 ºC for 6 days, as derived from HPLC/MS analysis in (b) negative ionization mode.

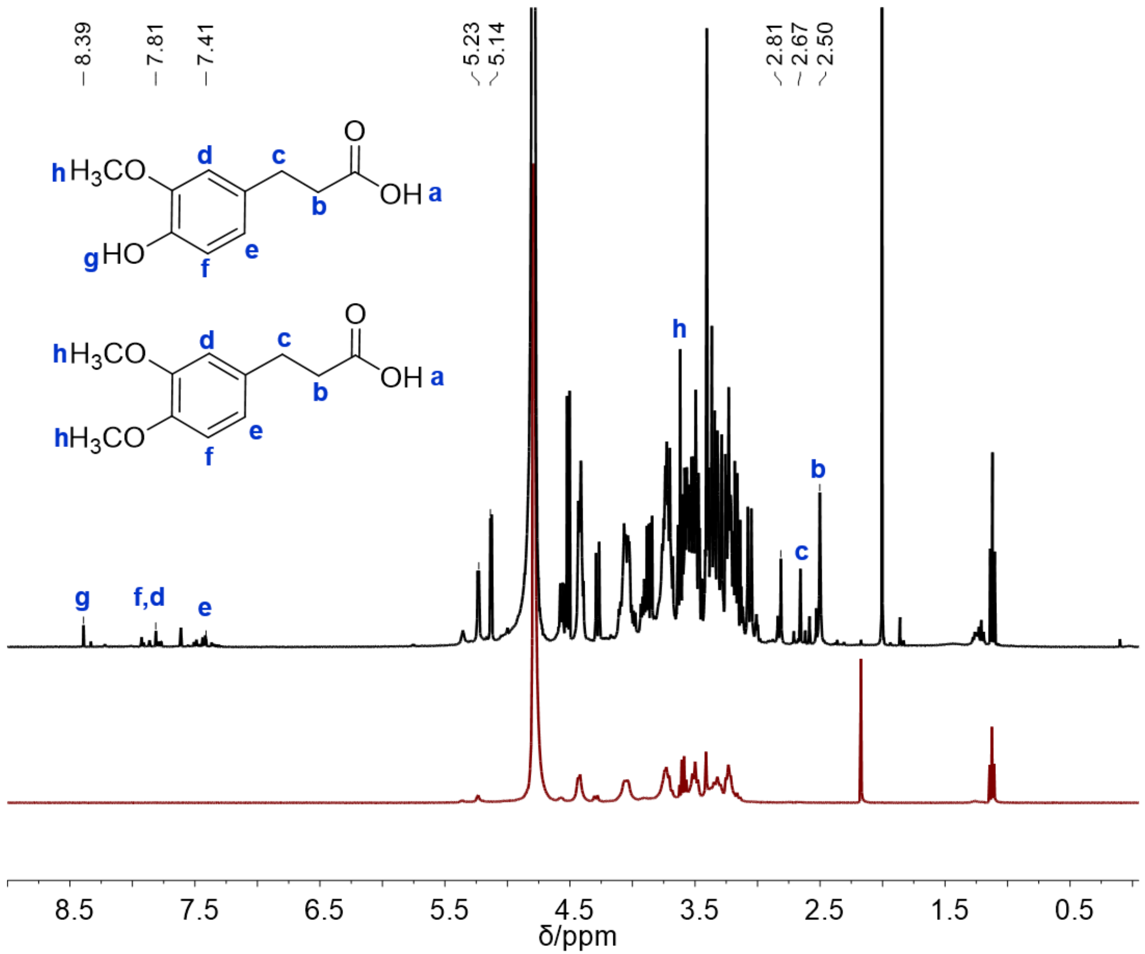

Figure S14. ^{1}H NMR spectrum in D_2O recorded after PR of xylan (black) with *α-cel*-CDs (2.2 mg) and **NiP** (50 nmol) in KP_i (pH 6) under simulated sunlight irradiation (AM 1.5 G, 100 mW cm^{-2}) at 25 ºC. For comparison the ^{1}H NMR spectrum of xylan before PR is shown in red. The chemical structures/shifts of the main $C_{10}H_{12}O_4$/$C_{11}H_{14}O_4$ PR products (δ = 2.50, 2.67, 3.8, 7.4, 7.81 and 8.5 ppm) are shown.

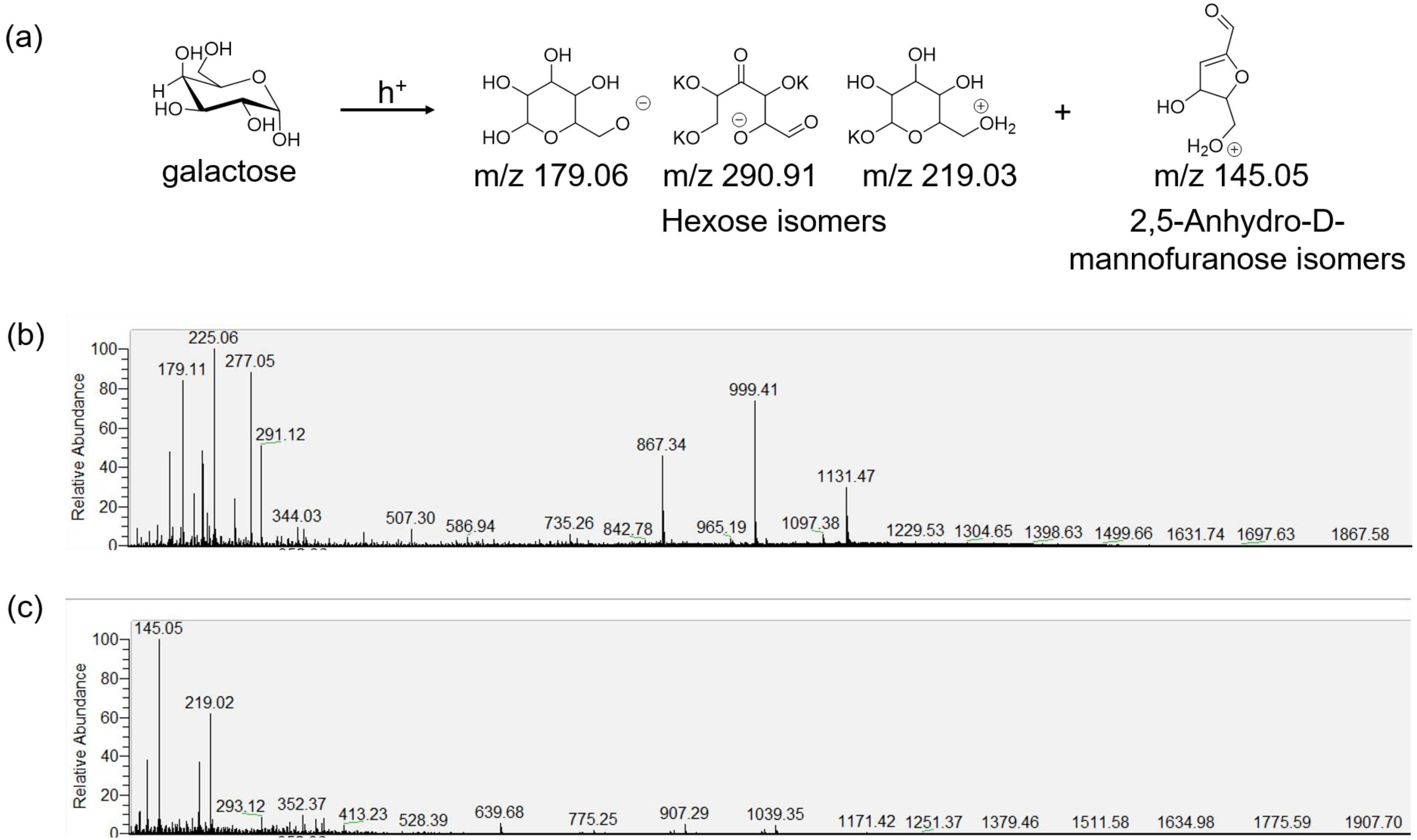

Figure S15. (a) Oxidation products from PR of galactose with *α-cel*-CDs (2.2 mg) and **NiP** (50 nmol) in KP_i (pH 6) under simulated sunlight irradiation (AM 1.5 G, 100 mW cm^{-2}) at 25 ºC for 6 days, as derived from HPLC/MS analysis in (b) negative and (c) positive ionization modes.

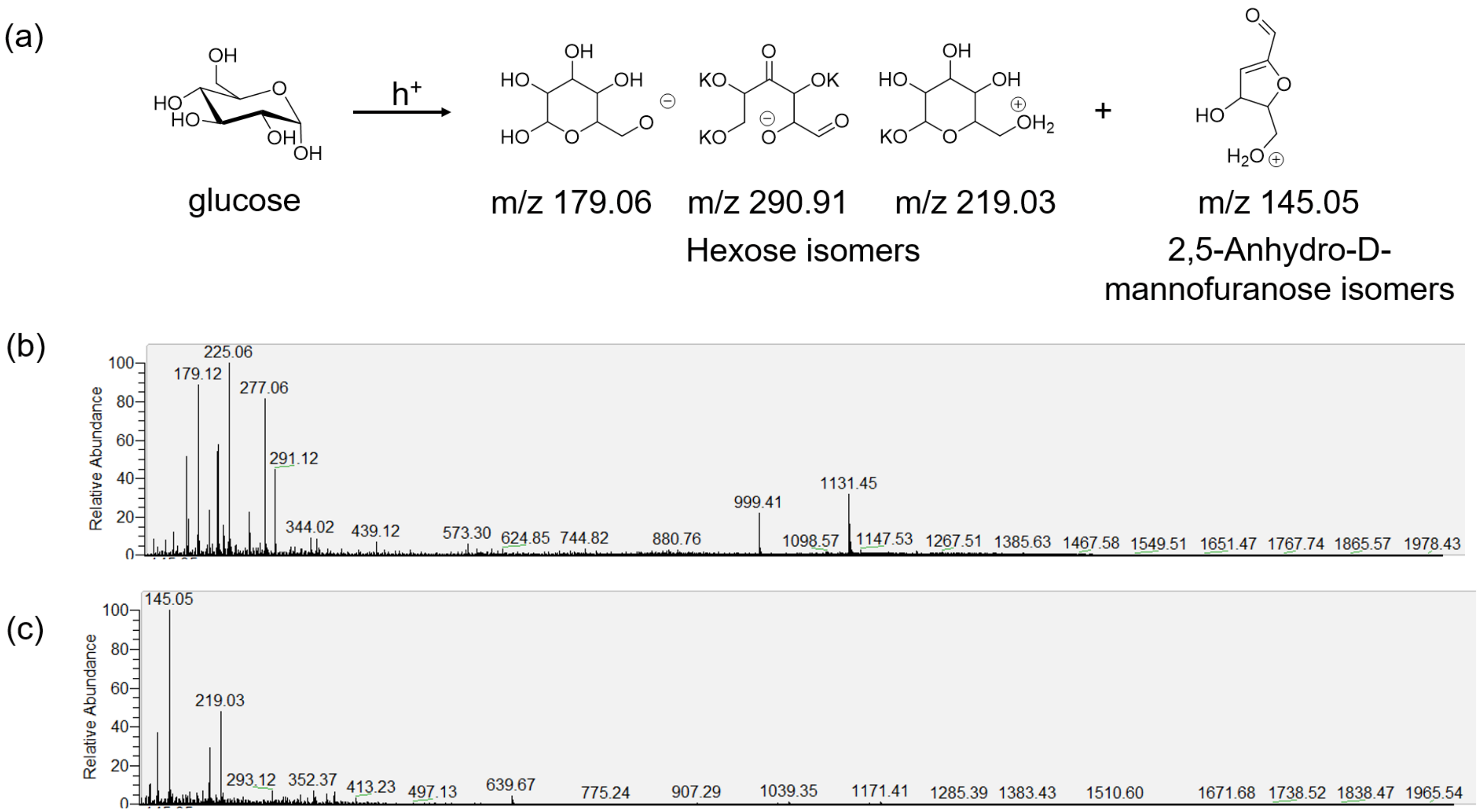

Figure S16. (a) Oxidation products from PR of glucose with *α-cel*-CDs (2.2 mg) and **NiP** (50 nmol) in KP_i (pH 6) under simulated sunlight irradiation (AM 1.5 G, 100 mW cm^{-2}) at 25 ºC for 6 days, as derived from HPLC/MS analysis in (b) negative and (c) positive ionization modes.

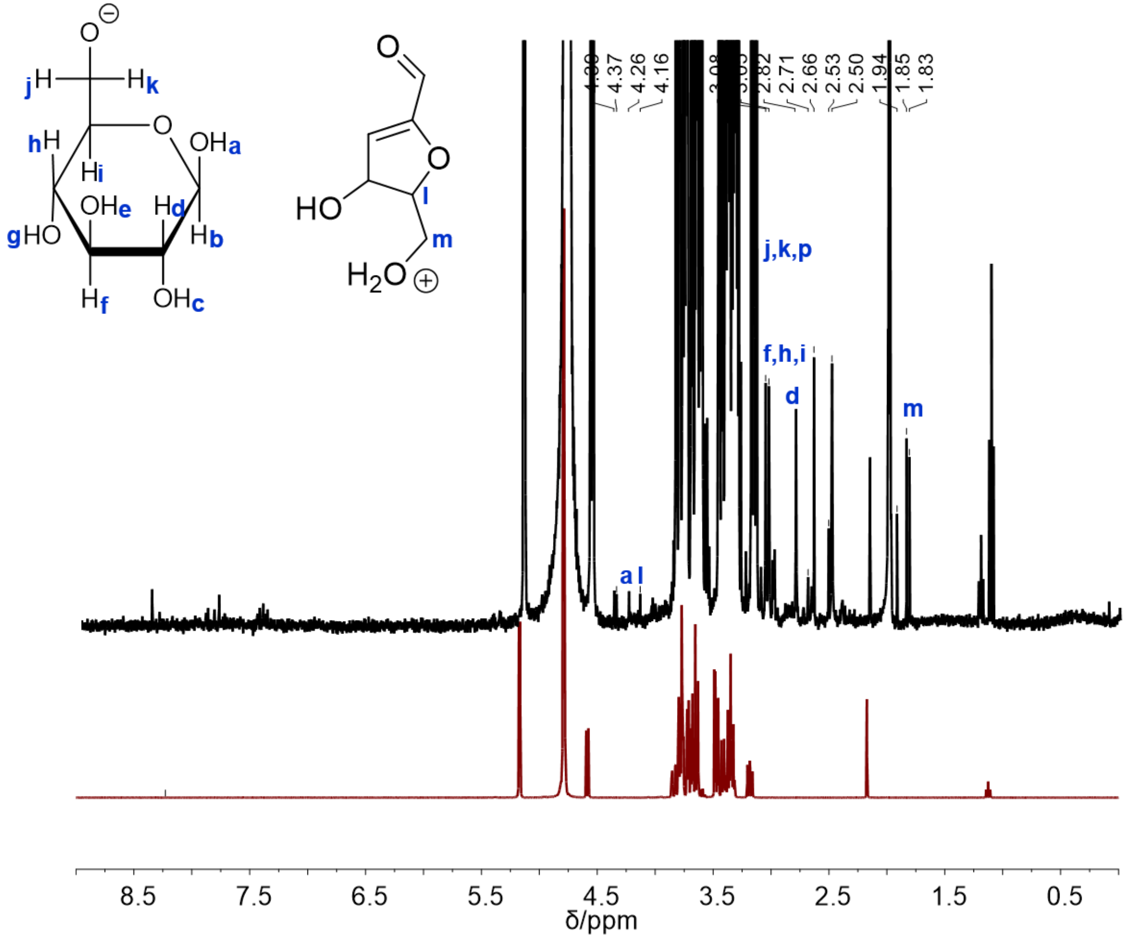

Figure S17. ^{1}H NMR spectrum in D_2O recorded after PR of glucose (black) with *α-cel*-CDs (2.2 mg) and **NiP** (50 nmol) in KP_i (pH 6) under simulated sunlight irradiation (AM 1.5 G, 100 mW cm^{-2}) at 25 ºC. The ^{1}H NMR spectrum of glucose before PR is shown in red. The chemical structures/shifts of the main $C_6H_{12}O_6$ (δ = 2.82, 3.03, 3.08, 3.20-3.90, 4.26 ppm) and $C_6H_{10}O_5$ oxidation products (δ = 1.83, 1.85, 4.16 ppm), along with their characteristic chemical shifts are also displayed.

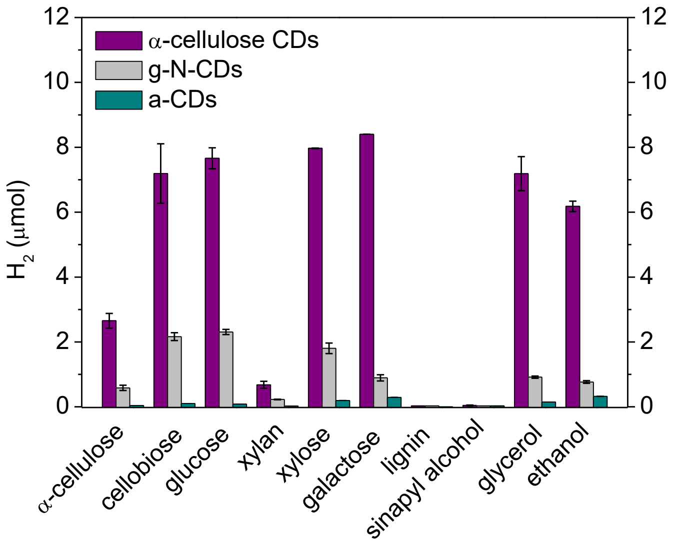

Figure S18. H_2 yields from PR with *α-cel*-CDs, *g*-N-CDs and *a*-CDs under real-world conditions, using untreated sea water (adjusted pH = 6) and biomass EDs (100 mg). All experiments were carried out under simulated sunlight irradiation (AM 1.5 G, 100 mW cm^{-2}) at 25 ^{o}C in the presence of **NiP** (50 nmol) for 24 h.

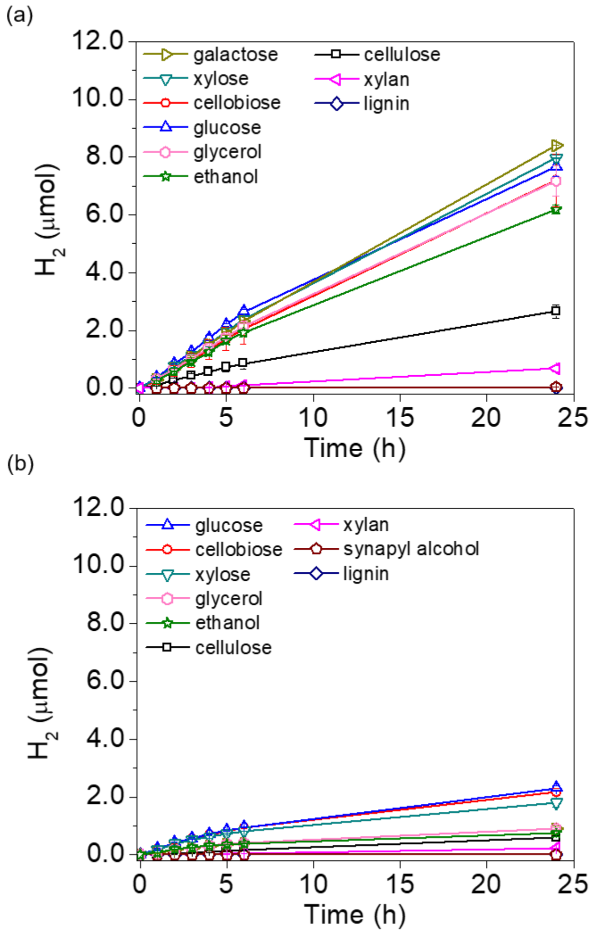

Figure S19. Photocatalytic H_2 evolution using (a) *α-cel*-CDs and (b) *g*-N-CDs in untreated sea water (adjusted pH = 6). Pure lignocellulosic components (100 mg) and biomass-derived compounds served as EDs. All experiments were carried out under simulated sunlight irradiation (AM 1.5 G, 100 mW cm^{-2}) at 25 ^{o}C for 24 h in the presence of **NiP** (50 nmol).

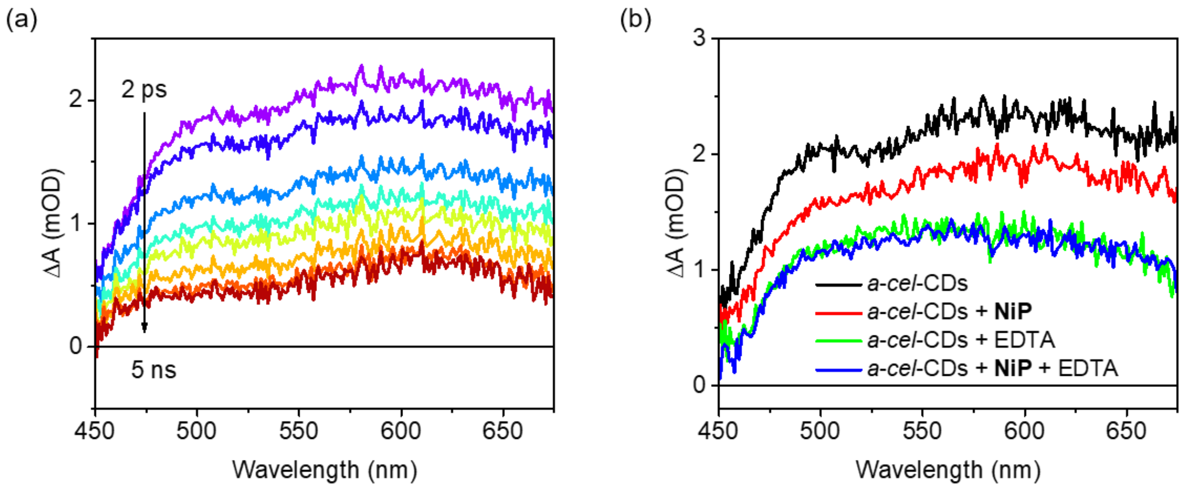

Figure S20. (a) fs-TA spectra of *α-cel*-CDs excited at 355 nm and sampled at delay times 2 ps−5 ns. (b) fs-TA spectra of *α-cel*-CDs measured at 0.5 ps in the presence of different photocatalytic components: *α-cel*-CDs only (black), in the presence of either **NiP** (red) or EDTA (blue), and with all photocatalytic components (green). Samples were excited at 355 nm with an energy of 256 μJ in KP_i buffer solution (pH 6) under Ar.

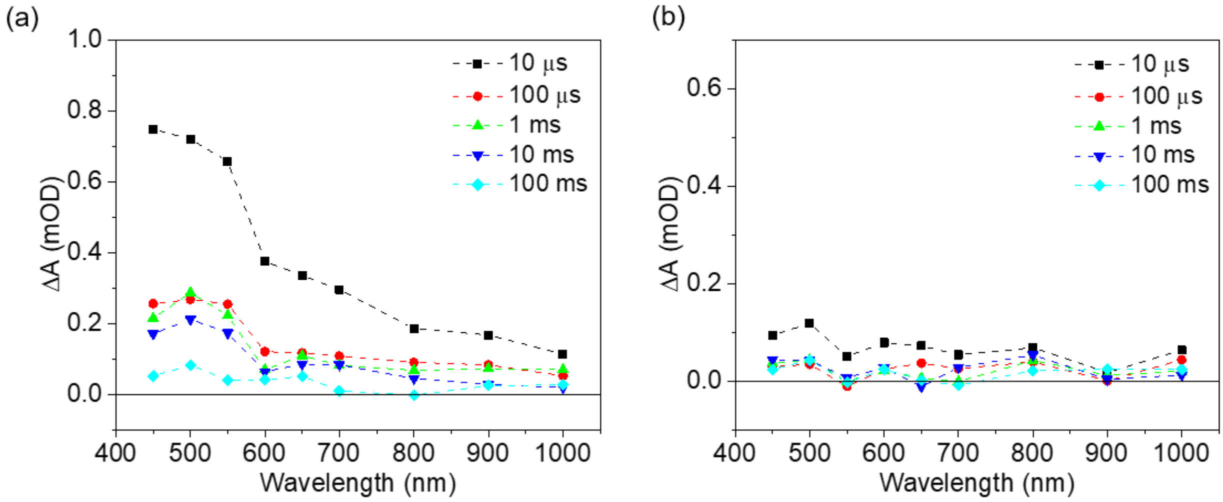

Figure S21. μs-TA spectra of *α-cel*-CDs excited at 355 nm and sampled at the indicated delay times; (a) under Argon and (b) under air (in the presence of O_2).

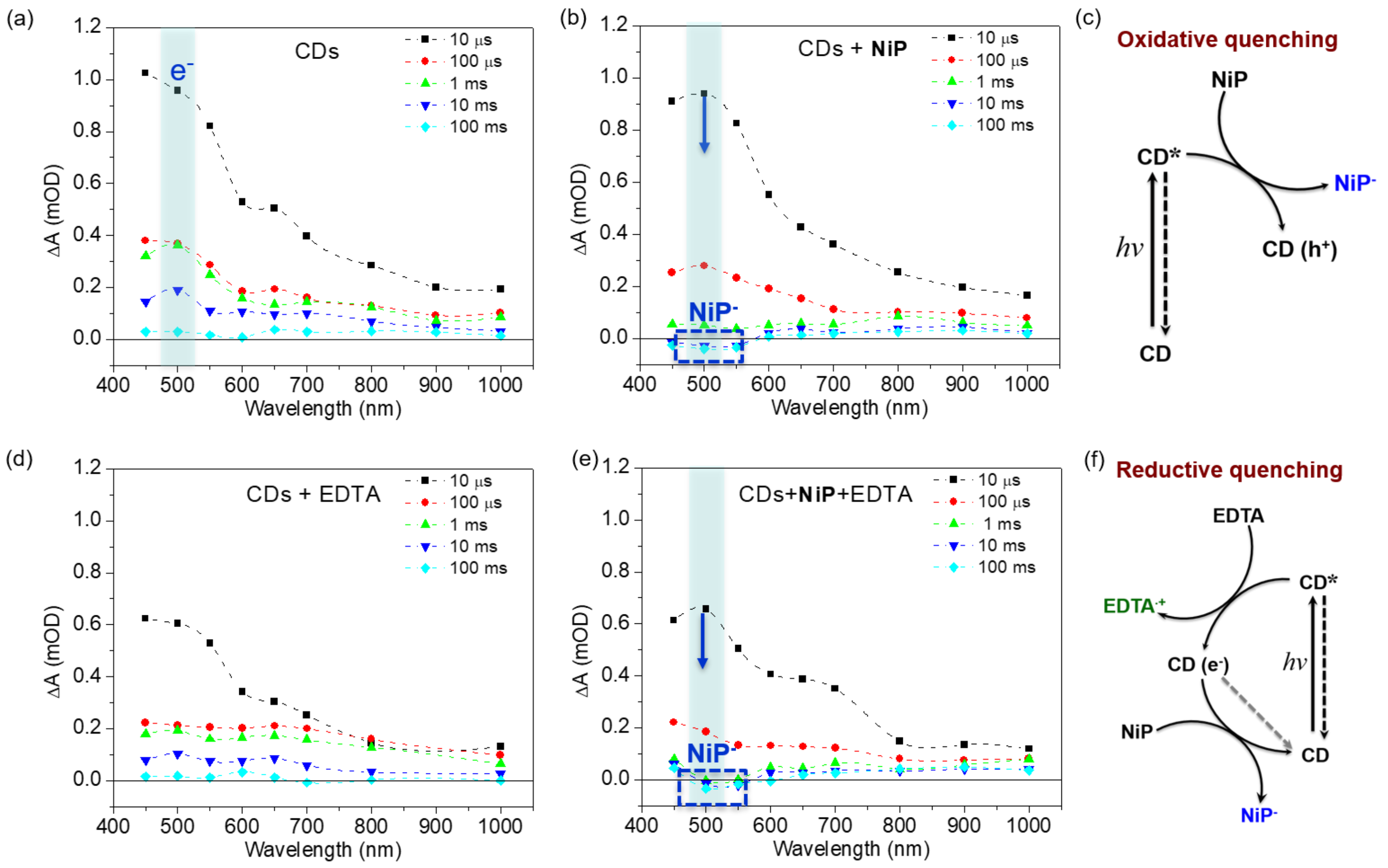

Figure S22. TA spectra of α-*cel*-CDs at different time delays under various conditions; (a) in the presence of only α-*cel*-CDs, (b) with α-*cel*-CDs and **NiP** but in the absence of EDTA, (c) schematic representation of oxidative quenching mechanism, (d) TA spectra of α-*cel*-CDs with EDTA in the absence of **NiP**, (e) with both EDTA and **NiP** and (f) schematic of reductive quenching mechanism.

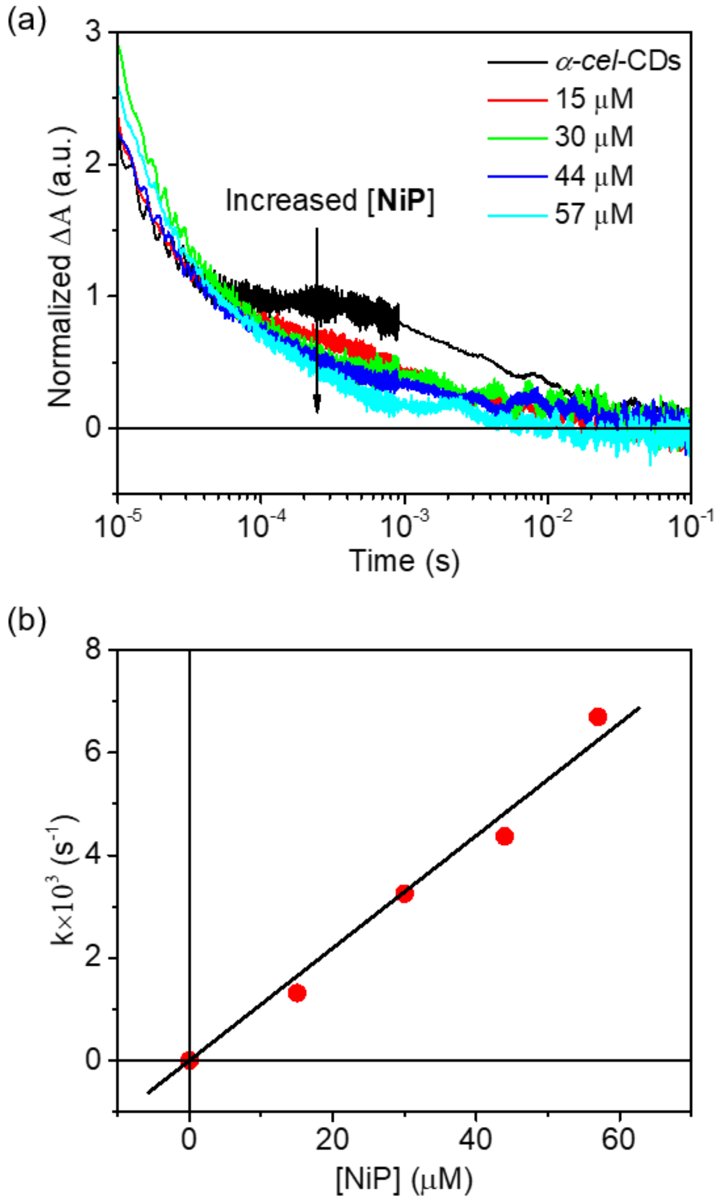

Figure S23. (a) Change of the decay kinetics of photogenerated electrons in *α-cel*-CDs due to the addition of **NiP**, (b) the change of the decay rate (characterized by the decay halftime) at 500 nm as a function of **NiP** concentration. Samples were excited at 355 nm with a pump power of 1 mJ cm^{-2} in KP_i buffer solution (pH 6) under Ar.

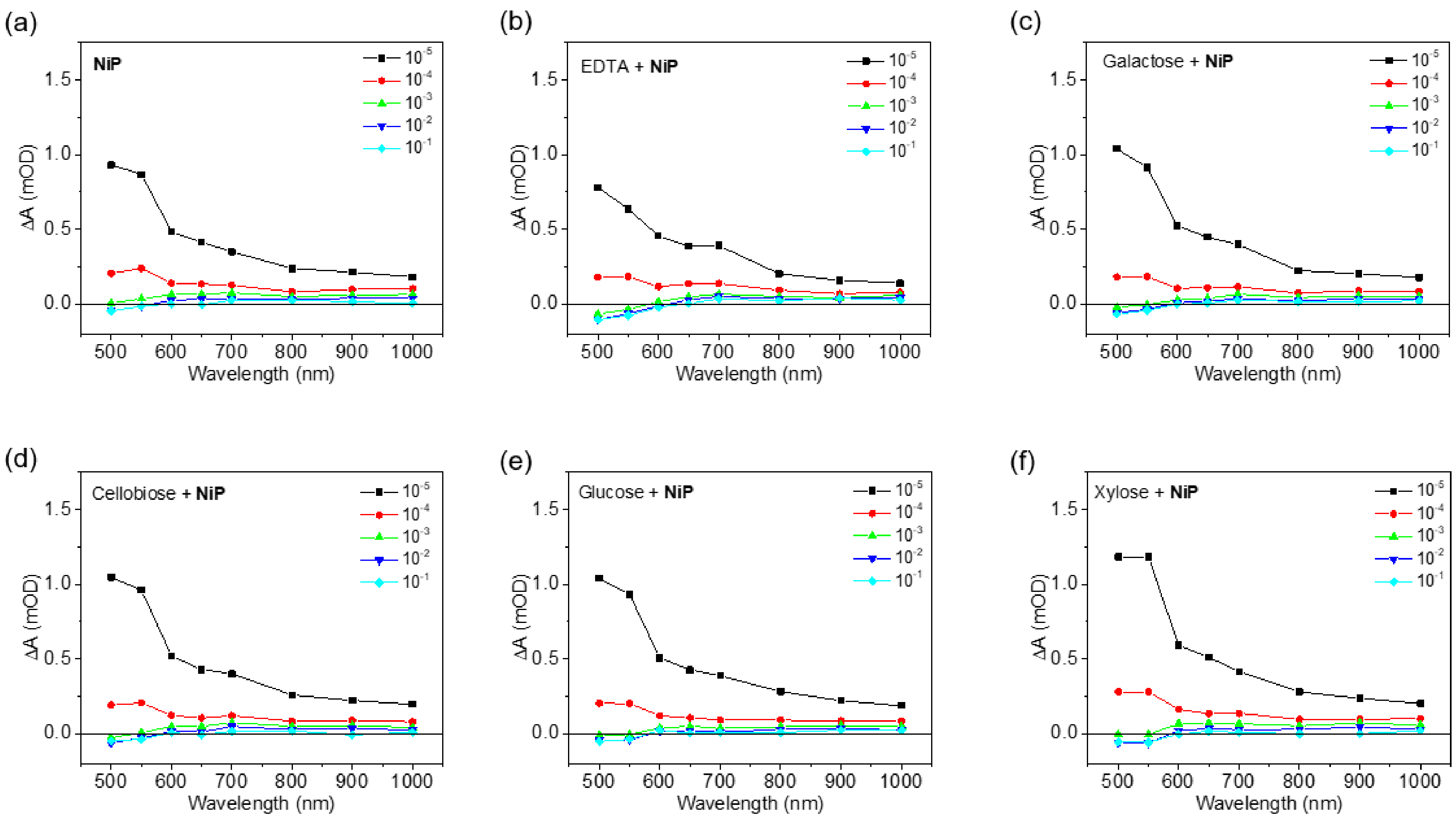

Figure S24. µs-TA spectra of *α-cel*-CDs before (a) and after the addition of different biomass substrates (0.1 M) as EDs; (b) EDTA, (c) galactose, (d) cellobiose, (e) glucose and (f) xylose. All experiments were carried out in KP_i (pH = 6.6), in the presence of **NiP** (50 nmol) upon excitation at 355 nm with an energy of 1 mJ cm^{-2}.

Supporting References

[S1] D. S. Achilleos, H. Kasap, E. Reisner, *Green Chem.* **2020**, *22*, 2831-2839.

[S2] B. C. M. Martindale, G. A. M. Hutton, C. A. Caputo, S. Prantl, R. Godin, J. R. Durrant, E. Reisner, *Angew. Chem. Int. Ed.* **2017**, *56*, 6459-6463.

[S3] B. C. M. Martindale, G. A. M. Hutton, C. A. Caputo, E. Reisner, *J. Am. Chem. Soc.* **2015**, *137*, 6018-6025.

[S4] M. A. Gross, A. Reynal, J. R. Durrant, E. Reisner, *J. Am. Chem. Soc.* **2014**, *136*, 356-366.

[S5] B. C. M. Martindale, E. Joliat, C. Bachmann, R. Alberto, E. Reisner, *Angew. Chem. Int. Ed.* **2016**, *55*, 9402-9406.

[S6] P. Yang, J. Zhao, J. Wang, H. Cui, L. Li, Z. Zhu, *ChemPhysChem* **2015**, *16*, 3058-3063.

[S7] C. A. Caputo, M. A. Gross, V. W. Lau, C. Cavazza, B. V. Lotsch, E. Reisner, *Angew. Chem. Int. Ed.* **2014**, *53*, 11538-11542.

[S8] H. Kasap, C. A. Caputo, B. C. M. Martindale, R. Godin, V. W.-h. Lau, B. V. Lotsch, J. R. Durrant, E. Reisner, *J. Am. Chem. Soc.* **2016**, *138*, 9183-9192.

[S9] H. Kasap, D. S. Achilleos, A. Huang, E. Reisner, *J. Am. Chem. Soc.* **2018**, *140*, 11604-11607.

[S10] D. J. Martin, K. Qiu, S. A. Shevlin, A. D. Handoko, X. Chen, Z. Guo, J. Tang, *Angew. Chem. Int. Ed.* **2014**, *53*, 9240-9245.

[S11] a) M. F. Kuehnel, E. Reisner, *Angew. Chem. Int. Ed.* **2018**, *57*, 3290-3296; b) G. Zhang, C. Ni, X. Huang, A. Welgamage, L. A. Lawton, P. K. J. Robertson, J. T. S. Irvine, *Chem. Commun.* **2016**, *52*, 1673-1676.

End of Supporting Information
